# Synthesis, Crystal Structure, and Biological Evaluation of Fused Thiazolo[3,2-*a*]Pyrimidines as New Acetylcholinesterase Inhibitors

**DOI:** 10.3390/molecules24122306

**Published:** 2019-06-21

**Authors:** Mohamed Y. Mahgoub, Awatef M. Elmaghraby, Abd-Elfttah A. Harb, João L. Ferreira da Silva, Gonçalo C. Justino, M. Matilde Marques

**Affiliations:** 1 Chemistry Department, Faculty of Science, South Valley University, Qena 83523, Egypt; m.mahgoub2013@gmail.com (M.Y.M.); alifaragharb@gmail.com (A.-E.A.H.); 2 Centro de Química Estrutural, Instituto Superior Técnico, Universidade de Lisboa, 1049-001 Lisboa, Portugal; joao.luis@tecnico.ulisboa.pt (J.L.F.d.S.); goncalo.justino@tecnico.ulisboa.pt (G.C.J.); matilde.marques@tecnico.ulisboa.pt (M.M.M.)

**Keywords:** thiazolopyrimidines, acetylcholinesterase inhibition, molecular docking, crystal structures

## Abstract

A new series of thiazolo[3,2-*a*]pyrimidine bromide salt derivatives **7a**–**d** were synthesized from 3,4-dihydropyrimidinethione precursors. The target compounds were fully characterized by 1D- and 2D-NMR, high resolution ESI-MS/MS and single crystal X-ray diffraction analysis, which confirmed a regioselective 5*H* cyclization of the dihydropyrimidinethiones. All target compounds were evaluated in vitro as human acetylcholinesterase (*h*AChE) inhibitors via an Ellman-based colorimetric assay and showed good inhibition activities (better than 70% at 10 µM and IC_50_ values in the 1 µM range). Molecular docking simulations for all target products into *h*AChE were performed and confirmed strong binding to the enzyme. These results provide a promising and new starting point to improve acetylcholinesterase inhibitors and explore novel treatment options against Alzheimer’s disease.

## 1. Introduction

**Scheme 1:**
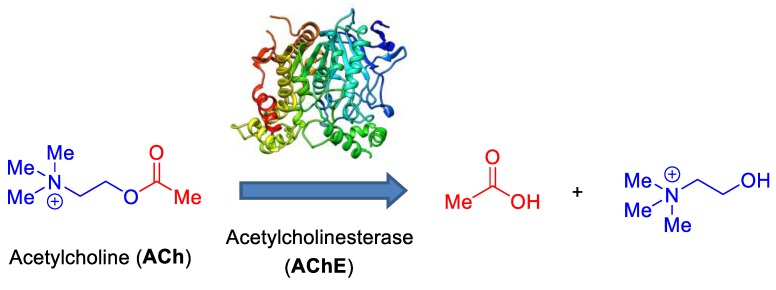
The hydrolysis of acetylcholine (ACh) by acetylcholinesterase (AChE) into acetic acid and choline.

Alzheimer’s disease (AD) is a chronic and progressive neurodegenerative disorder that is the leading cause of dementia in elderly people, accounting for 60–80% of all cases. According to the World Health Organization (WHO), AD affected about 50 million people worldwide in 2018 [1]. AD destroys brain cells and nerves, disrupting the neurotransmitters that carry messages in the brain, especially those responsible for storing memories. Acetylcholinesterase (AChE) is the enzyme that catalyzes the hydrolysis of the neurotransmitter acetylcholine (ACh) into acetic acid and choline (Scheme 1). According to the cholinergic hypothesis, depleted levels of ACh are associated with AD [2,3].

**Figure 1:**
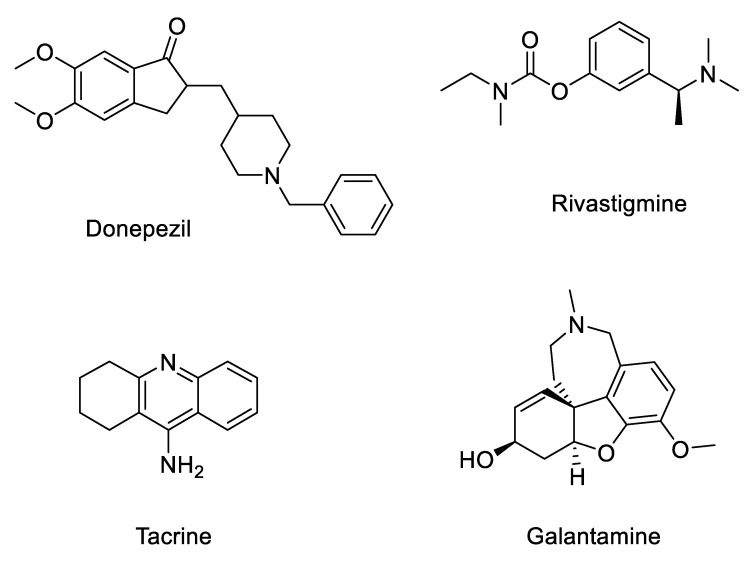
Structures of approved AChE inhibitors.

As a result, AChE inhibitors, such as tacrine, donepezil, galantamine, and rivastigmine (Figure 1), represent the major class of drugs approved and prescribed for AD. These drugs promote memory function and delay the cognitive decline, although they do not change the underlying pathology [4].

Thiazolopyrimidine derivatives have been the focus of intense research in recent years. Among the various members of this class, thiazolo[3,2-*a*]pyrimidines are particularly interesting. They have been reported to inhibit specific enzymes, such as AChE [5], cell division cycle (Cdc) 25B phosphatase [6], casein kinase 2 [7], YycG histidine kinase [8], 2-methylerythritol 2,4-cyclodiphosphate synthase (IspF) proteins from *Mycobacterium tuberculosis*, *Plasmodium falciparum*, and *Arabidopsis thaliana* [9], and xanthine oxidase [10]. While the precise biological targets remain to be elucidated in some cases, thiazolo[3,2-*a*]pyrimidines have widely been reported to possess anti-cancer [11], anti-inflammatory and analgesic [12,13,14,15], anti-microbial, antibiofilm, antioxidant, DNA cleavage [8,16,17,18,19,20], antiviral [21,22], anti-Parkinsonian [23], antidiabetic [24], antihypertensive, and inotropic [25] activities. Other possible applications include their use as antimalarials and inhibitors of HIV reverse transcriptase [9,26], as well as antagonists of the 5-HT2 serotonin receptor, with potential interest for the treatment of depression [27,28]. Other non-therapeutic uses for thiazolo[3,2-*a*]pyrimidines include inhibition of mild steel corrosion [29], and dimeric thiazolo[3,2-*a*]pyrimidinones have been proposed for the development of pigments, sensitizers in solar cells, and molecular switches [30]. As part of a program aiming to obtain new potent AD modifying agents, we report herein a new small series of AChE inhibitors based on a fused thiazolopyrimidine core. The plausible binding interactions of two representative compounds at the active site of human AChE are also discussed.

## 2. Results and Discussion

### 2.1. Synthesis

**Scheme 2:**
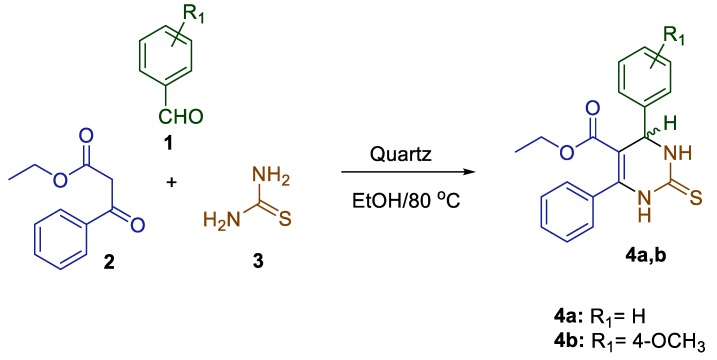
Synthesis of 3,4-dihydropyrimidinethiones (DHPMs) **4a**,**b** by a multicomponent Biginelli-type reaction.

**Scheme 3:**
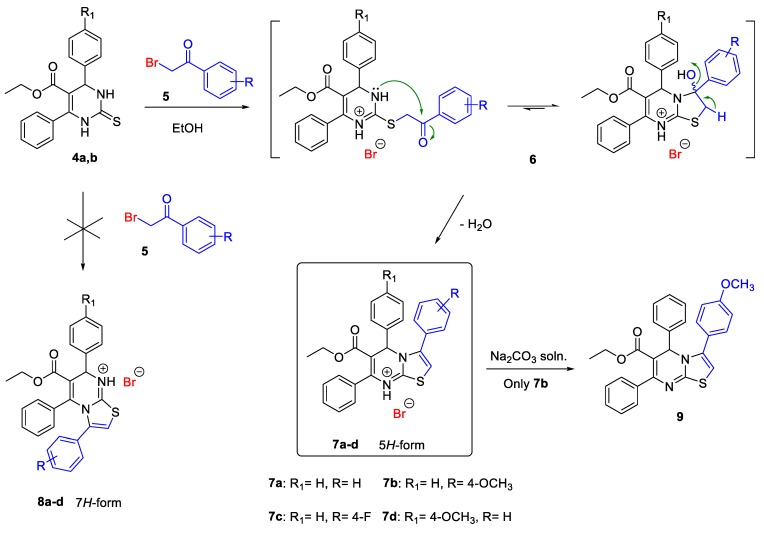
Synthesis of fused thiazolo[3,2-*a*]pyrimidines **7a**–**d** and **9.**

Herein, a new series of thiazolo[3,2-*a*]pyrimidines **7a**–**d**, were designed and synthesized. Initially, 3,4-dihydropyrimidinethiones (DHPMs) **4a**,**b** were prepared by a multicomponent Biginelli-type reaction based on condensation of aldehydes **1**, ethyl benzoylacetate **2** and thiourea **3**, as previously described [31] (Scheme 2). Then, a series of fused thiazolo[3,2-*a*]pyrimidines **7a**–**d** were synthesized in good yields, in a regioselective manner, through the intermediate **6** by cyclization of DHPMs **4a**,**b** with phenacyl bromides **5** under refluxing conditions in ethanol for 12–15 h (Scheme 3). The bromide salts were used for biological activity measurements. For comparative characterization purposes, compound **7b** was also neutralized to the corresponding product **9.**

### 2.2. Structural Characterization

The DHPMs **4a**,**b** and the thiazolo[3,2-*a*]pyrimidine salts **7a**–**d** were fully characterized by 1D- and 2D-NMR, high resolution mass spectrometry (HRMS), and single crystal X-ray diffraction analysis.

#### 2.2.1. NMR Data

The NMR spectra were fully consistent with the expected structures. The most characteristic features in the NMR spectra of DHPMs **4a**,**b** were the methine proton and carbon shifts, observed at δ 5.27 and 54.08 ppm, respectively, in **4a** and at δ 5.22 and 53.52 ppm, respectively, in **4b**. Additionally, the two exchangeable NHs were observed at downfield shifts around δ 9.7 ppm for N3-**H** and δ10.4 ppm for N1-**H**. Using a HMBC pulse sequence, 2- and 3-bond correlations were observed between the methine proton and nearby carbons (C=S, C=O, C5, and C6), thus confirming the structural characterization.

Theoretically, the intramolecular cyclization of DHPMs with phenacyl bromide derivatives might afford two possible isomeric products: 5*H*-thiazolo[3,2-*a*]pyrimidines **7a**–**d** and 7*H*-thiazolo[3,2-*a*]pyrimidines **8a**–**d**. However, a single product was obtained in all instances, presumably due to the higher conjugation (and thereby increased contribution) of the tautomer shown in Scheme 3 for the non-fused precursor **6**, compared to the other possible tautomeric form (not shown). Cyclization was confirmed by the absence of thioamide NH resonances in the NMR spectra of **7a**–**d**, along with the presence of an aromatic proton singlet (C2**-**H) at around δ 7.15–7.30 ppm and the corresponding C2 resonance at δ 113.00–114.60 ppm. Moreover, compared with **4a**,**b**, the further downfield shifts observed for the methine proton and carbon resonances upon cyclization (Table 1) were consistent with formation of the *5H* isomers **7a**–**d**. This was further corroborated by single crystal X-ray diffraction characterization (*cf.* paragraph below). Neutralization of **7b** yielded compound **9**, which had characteristic proton (C2-H) and carbon (C2) resonances at δ 6.20 and 103.04 ppm, respectively, slightly upfield from those of **7b**, as expected (*cf.* experimental section and Supplementary data).

**Figure 2:**
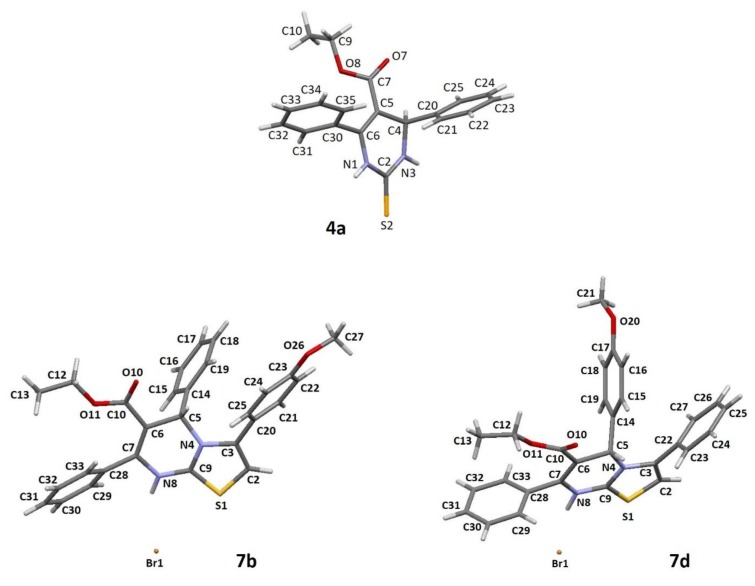
Diagrams of the molecular structures of compounds **4a**, **7b**, and **7d** showing the atomic labelling scheme.

For comparative purposes, the relevant carbon chemical shifts of compounds **7b** and **9** are listed in Table 2. The most notable changes were observed for C2, C7, and C9, which is consistent with protonation of N8 in compound **7b**. This assumption was further confirmed by single crystal X-ray diffraction characterization (*cf.* Figure 2).

#### 2.2.2. X-Ray Diffraction Studies

Single crystal X-ray diffraction analyses allowed us to confirm the structures of compounds **4a**, **7b**, **7d** without ambiguity (Figure 2). Some of the relevant crystal parameters are reported in Table S1, in the Supplementary data section, while selected geometrical parameters are presented in Table 3 and Table 4. Structurally-comparable bond lengths and angles were similar in all molecules and well within the expected range, as judged from extensive analysis of the values included in the CSD [32,33] (see also Tables S2 and S3, in the Supplementary data section, for full geometric parameters). All compounds had phenyl groups bonded to a quasi-planar dihydropyrimidine-containing central ring (Figure 2 and Table 3 and Table 4) displaying very similar angles between the planes. Another fragment present in all compounds (**4a**, **7b**, **and 7d**) was the ester group, with torsion angle values 11.5°, 21.9°, and 32.7°, respectively (Table 3 and Table 4). Despite these slight differences, they all presented similar packing index values; in the absence of any stereochemical constraint it is possible to attribute the main geometrical features in these compounds to an attempt of achieving the most compact structures possible [34].

Concerning compound **4a** it was clear that S(2)-C(2) has all the characteristics of a double bond: angles around C(2) in the 116.4–121.9° range and a bond length of 1.680 Å; C=S double bonds usually display values between 1.630 and 1.720 Å, while single bonds are longer, as expected (1.749–1.856 Å) [32].

In compounds **7b** and **7d** there was a *Z* C(2)-C(3) double bond, with bond lengths of 1.335 and 1.339 Å, respectively. Moreover, the bond length values for S(1)-C(2) and S(1)-C(9) in both compounds suggest an extended delocalization spanning the sulfur atom.

### 2.3. Supramolecular Interactions

#### 2.3.1. Hirshfeld Surface Analysis

The 3D normalized contact distance (*d*_norm_) Hirshfeld surface map for compounds **4a**, **7b**, and **7d** was generated using the Crystal Explorer 3.1 software [35] to investigate the intermolecular interactions in the crystal lattices. This parameter is defined as

**Figure 3:**
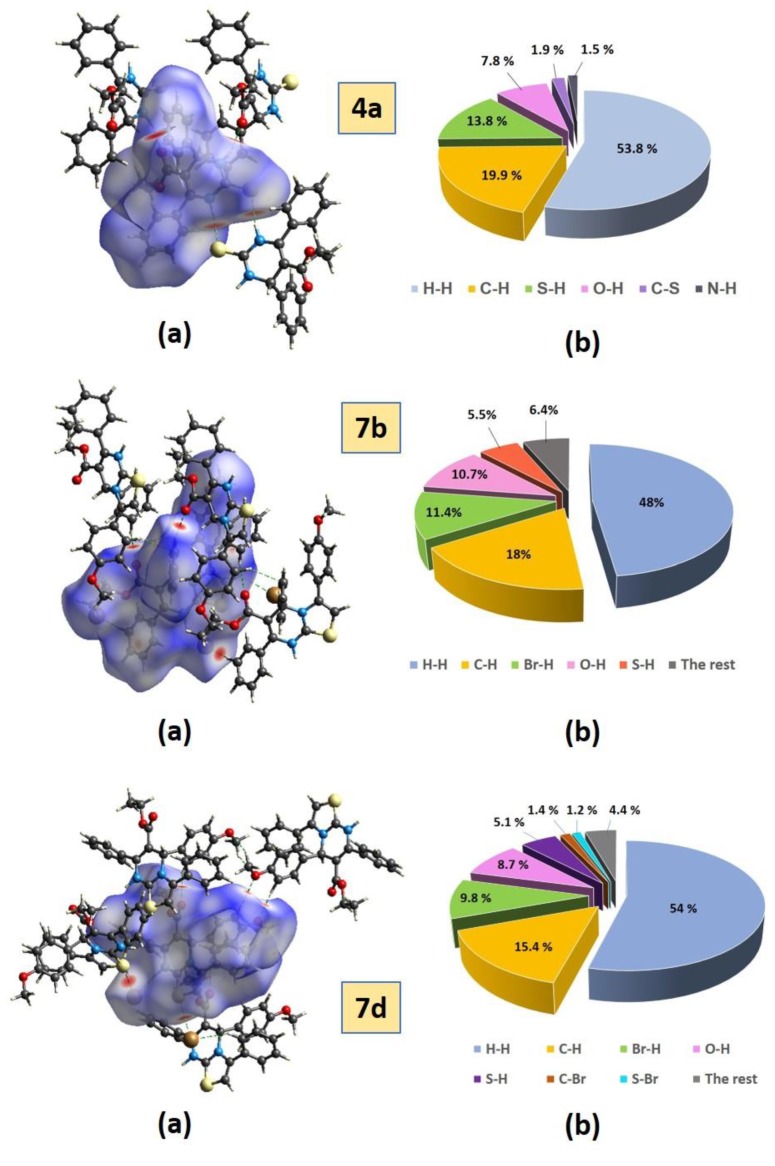
(**a**) 3D Hirshfeld surfaces mapped with *d*_norm_; red colored regions show shorter contacts; (**b**) histograms showing the percentage contributions of the various types of interactions predicted for compounds **4a**, **7b**, and **7d.**

*d*_norm_ = (*d*_i_ − *r*_i_^vdw^)/*r*_i_^vdw^ + (*d*_e_ − *r*_e_^vdw^)/*r*_e_^vdw^

where *r*_i_^vdw^ and *r*_e_^vdw^ are the van der Waals radii of the atoms involved, *d*_e_ represents the distance from the Hirshfeld surface to the nearest atom external to the surface, and *d*_i_ represents the distance from the Hirshfeld surface to the nearest atom internal to the surface [36,37]. The value of *d*_norm_ is positive or negative when intermolecular contacts are longer or shorter than *r*^vdw^, respectively. The Hirshfeld surface map uses a coloring code to represent the proximity of intermolecular interactions around the molecule within a network: white for *d* around the van der Waals distance, blue for *d* longer than van der Waals distance, and red for *d* shorter than the van der Waals distance [38]. The 3D *d*_norm_ Hirshfeld surface plots of the selected compounds **4a**, **7b**, and **7d** included in this study are shown in Figure 3a. The plots show the main different intermolecular interactions present in each molecule. The large and deep red spots on the 3D Hirshfeld surfaces indicate the mainly strong close-contact hydrogen-bonding interactions. Additionally, the presence of weak hydrogen bonds is predicted in all molecules, involving C-H^…^X (X = O, N, S, Br) interactions. Other possible intermolecular interactions appear as lighter red regions (Figure 3a). The combination of *d*_e_ and *d*_i_ in the form of two-dimensional (2D) fingerprint plots provides a summary of the intermolecular contacts in the crystal [37]. Thus, the histograms in Figure 3b present the summary of the percentage contributions of the various types of potential interactions. For all compounds, the major contribution was related to the proximity-induced (H^…^H) (48–54%) and (C^…^H) interactions (15.4–19.9%) (Figure 3b). The major contacts expected in compound **4a** were N-H^…^S (13.8%) and N-H^…^O (7.8%). Noteworthy, in the case of compounds **7b** and **7d**, was the prediction of strong Br^-…^H8-N8 hydrogen bond contributions (11.4 and 9.8%, respectively), together with weak C-H^…^O hydrogen bonds (10.7 and 8.7%, respectively) (Figure 3b).

#### 2.3.2. Crystal Packings

Analysis of the crystal packing for these compounds confirmed that they are all racemic mixtures, as expected from the lack of stereoselectivity of the reaction conditions involved in their synthesis. The interactions detected on each compound using Platon and visualized using Mercury were consistent with the Hirshfeld analysis results.

**Figure 4:**
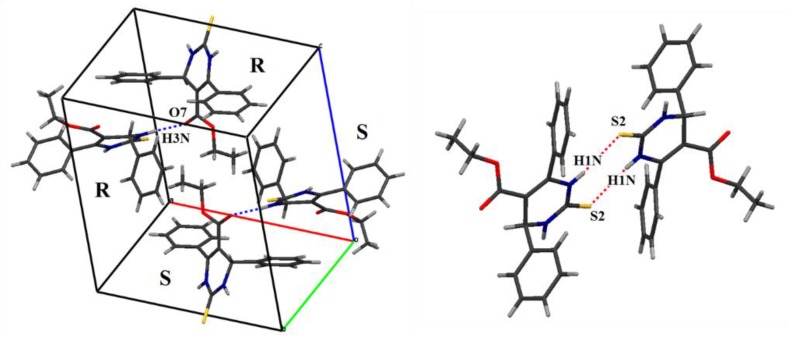
Hydrogen bonds in compound **4a**: Inside the unit cell (left) and with other molecules outside the unit cell (right).

Compound **4a** was found to have four molecules in the unit cell, two of isomer *R* and two of isomer *S*. The molecule has a good hydrogen donor system that formed classical hydrogen bonds: N(3)-H(3N)^...^O(7) between molecules inside the unit cell and an R^2^_2_(8) synthon involving N(1)-H(1N)^...^S(2) interactions with molecules outside the unit cell (Figure 4 and Table 5).

**Figure 5:**
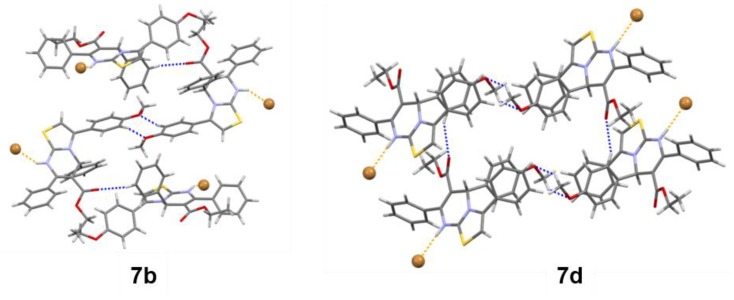
Main supramolecular interactions in compounds **7b** and **7d** (classical hydrogen bonds are represented in orange, weak hydrogen bonds in blue).

Due to the presence of Br^-^ ions, the crystal structure of compound **7d** was dominated by the strong anion^…^H-N hydrogen bond with the protonated N8, but it also included weak C-H^…^O hydrogen bonds, one of them (C21-H21C^...^O20) forming an R^2^_2_(6) synthon between molecules of the two stereoisomers. A similarly strong Br^-…^H8-N8 hydrogen bond was formed in compound **7b**, in which case the anion was also involved in a weak hydrogen bond, acting as a bridge between two cations of the same enantiomer. R^2^_2_(8) synthons were formed by C-H^…^O hydrogen bonds between *R-* and *S-* molecules (Figure 5 and Table 5).

### 2.4. Molecular Docking Studies to Human Acetylcholinesterase

**Figure 6:**
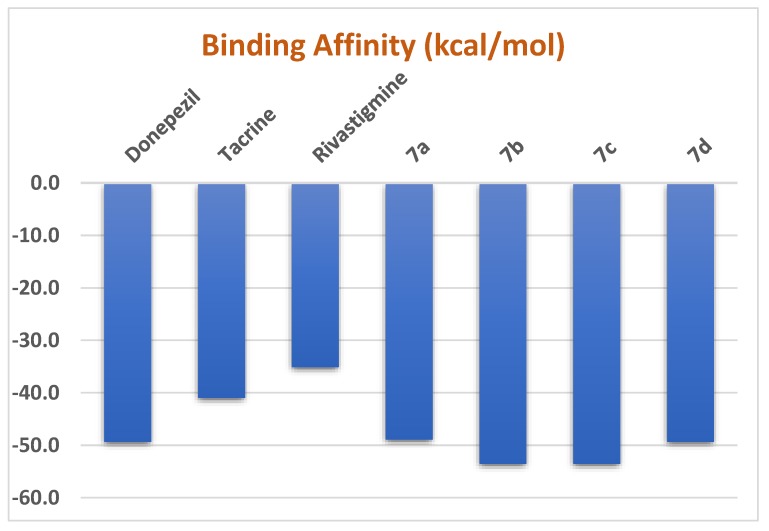
Comparison of the binding free energies of compounds **7a**–**d** and the reference *h*AChE inhibitors donepezil, tacrine, and rivastigmine to the active site gorge of *h*AChE.

In order to investigate the affinities of the fused thiazolo[3,2-*a*]pyrimidines **7a**–**d** toward human acetylcholinesterase *(h*AChE), we started by performing molecular docking simulations of the target compounds into the active site gorge of *h*AChE (PDB ID: 4 m0f, chain A), using Autodock Vina software [39]. Docking simulations were performed concomitantly with the known *h*AChE inhibitors, donepezil, tacrine, and rivastigmine, used as reference drugs for comparative purposes. The results for the binding free energies of all compounds investigated are summarized in Figure 6.

**Figure 7:**
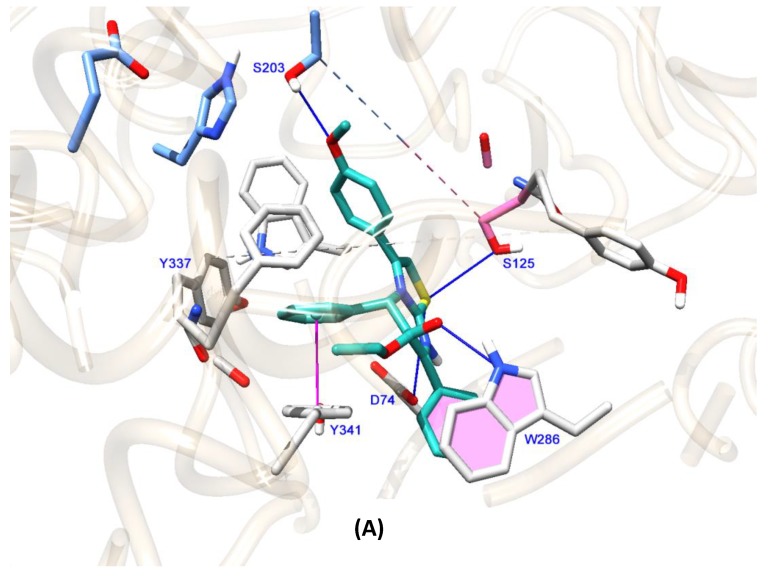

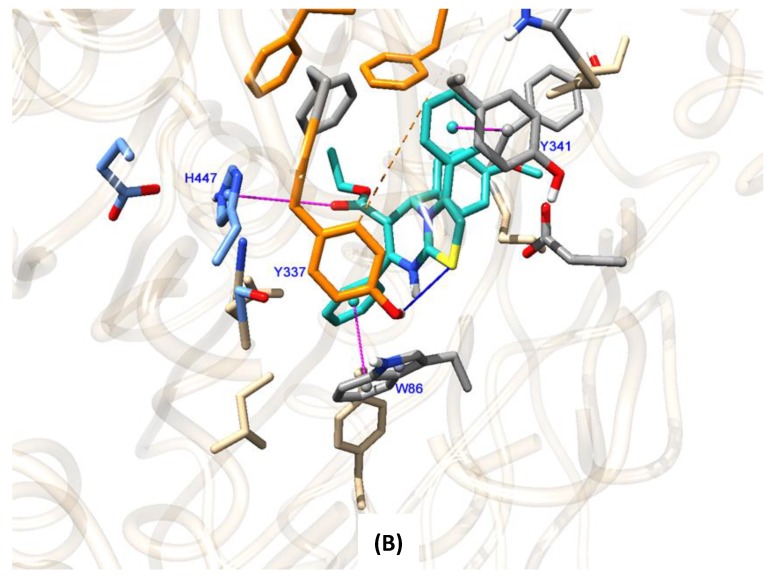
(**A**) and (**B**) A close view of **7b** and **7d** docked into the active site gorge of human AChE. Catalytic residues are shown in blue and the peripheral anionic site residues are shown in grey; the ligands are shown in light blue.

Molecular docking indicated good binding to *h*AChE, with all compounds **7a**–**d** binding equally or more strongly than any of the positive controls, which suggests that the compounds are potentially powerful AChE inhibitors. We further probed the structural features responsible for the strong binding to the enzyme. As representative examples, Figure 7A,B displays the docking poses for compounds **7b** and **7d** (*cf.* next paragraph).

A number of electrostatic interactions were found between **7b** and the side chain O atoms of Ser125 (at 4.0 Å) and of Asp74 (at 4.0 Å), and between the keto O atom of the ligand and the side chain N atom of Trp286 (at 3.0 Å). An H bond is also possible between the latter atom pair (with a H-acceptor distance of 2.1 Å and a donor-acceptor distance of 3.0 Å), albeit at a donor-H-acceptor angle on the lower limit of the allowed region (135°). Aromatic interactions are also quite evident between the ligand and nearby amino acid residues, in particular a π-π stacking involving the planes of the Trp286 side-chain and of one of the ligand aromatic rings (at a 3.9 Å distance, with an inter-plane angle of 9.3°). Another π-π stacking between the aromatic plane of Tyr341 and an aromatic plane of the ligand is also possible (at a 3.7 Å distance, with an inter-plane angle of 2.1°).

Concerning ligand **7d**, aromatic interactions are also evident between the ligand aromatic planes and the aromatic planes of Tyr341 (at a 4.3 Å) and Trp86 (at 4.3 Å); an anion-π interaction is also likely to occur between the carbonyl O atom of the ligand and the imidazole ring of His 447. Various electrostatic interactions are also possible between the ligand and protein amino acid residues, noticeably from the ligand S atom to the phenolic O atom of Tyr337 (at a 4.0 Å distance).

Although the poses differ between the compounds, the two panels in Figure 7 show both hydrophobic and hydrogen bond interactions with amino acids located in the peripheral anionic site (PAS) at the mouth of the gorge. PAS is a well-known substrate-binding site in AChE and binding of ligands at this location has been amply reported to affect the catalytic activity of the enzyme, by blocking access to the catalytic site and/or inducing an allosteric alteration of the catalytic triad conformation and efficiency [40,41,42].

### 2.5. In Vitro hAChE Inhibition

In view of the encouraging results obtained in the *in silico* study, compounds **7a–d** were evaluated for their *h*AChE inhibitory activity at up to 50 µM concentrations using a standard Ellman-based colorimetric protocol [43]. For comparative purposes, the powerful *h*AChE inhibitor methyl paraoxon was used as positive control.

**Figure 8:**
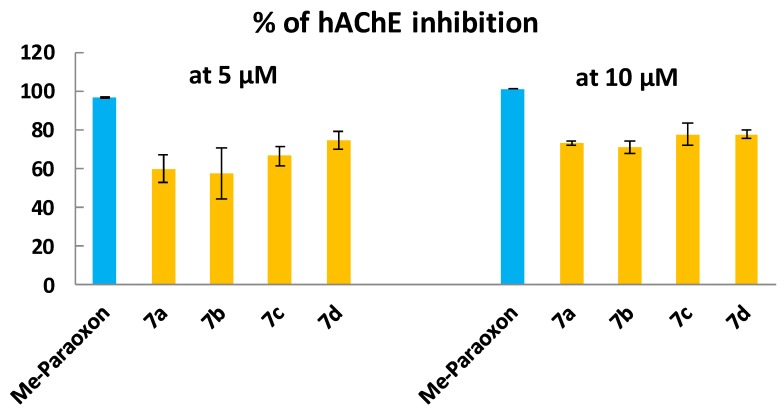
Experimental *in vitro h*AChE inhibition activities of the fused thiazolo[3,2-*a*]pyrimidines **7a**–**d** and the positive control, methyl paraoxon, at 5 and 10 µM. The data are expressed as average ± standard deviation of two independent experiments.

As shown in Figure 8, all compounds demostrated *h*AChE inhibitory activities at the micromolar (µM) level, ranging from 73–77.5% at 10 µM to 59–74% at 5 µM; at the same concentrations, the positive control inhibitor yielded approximately 100% inhibition. IC_50_ values for *h*AChE inhibition, obtained for compounds **7a**–**c**, were approximately 1 μM (**7a**, 1.59 μM; **7b**, 1.00 μM; **7c**, 1.17 μM), as compared to 4.9 nM for the positive control, methyl paraoxon, under the conditions of the assay. Likewise, *h*AChE inhibitors such as donepezil, rivastigmine, and tacrine are reported to have IC_50_ values in the low nanomolar range, under optimal assay conditions [44].

## 3. Materials and Methods

All reagents were purchased from commercial sources and used without further purification, unless stated otherwise. Whenever necessary, solvents were dried by standard methods [45]. Melting temperatures were measured with a Leica Galen III hot stage apparatus and are uncorrected.

^1^H-NMR spectra were recorded on a Bruker Avance III 400 spectrometer, operating at 400 MHz. ^13^C-NMR spectra were recorded on the same instrument, operating at 100.62 MHz. Chemical shifts are reported in ppm downfield from tetramethylsilane and coupling constants (*J*) are reported in Hz. Resonance and structural assignments (indicated using the IUPAC nomenclature system) were based on the analysis of coupling patterns, including the ^13^C-^1^H coupling profiles obtained from heteronuclear single quantum coherence (HSQC) and heteronuclear multiple bond correlation (HMBC) experiments, performed with standard pulse programs. The abbreviations Ph and Ar represent phenyl and aryl groups, respectively.

Low resolution mass spectra (MS) were recorded on an LCQ Fleet ion trap mass spectrometer equipped with an electrospray (ESI) ion source (Thermo Scientific). The mass spectrometer was operated in the ESI positive ion mode, with the following optimized parameters: Ion spray voltage, + 4.5 kV; capillary voltage, 16 V; tube lens offset, −63 V; sheath gas (N_2_), 80 arbitrary units; auxiliary gas (N_2_), five arbitrary units; capillary temperature, 300 °C. Spectra typically correspond to the average of 20−35 scans and were recorded in the range between 100 and 1500 Da. Tandem mass spectra (collision-induced dissociation experiments) were obtained with an isolation window of 4−9 Da, a 20−30% relative collision energy, and an activation energy of 30 ms. Data acquisition and processing were performed using the Xcalibur software.

High-resolution mass spectra (HRMS) were obtained on a Bruker Impact II quadrupole time-of-flight mass spectrometer (Bruker Daltoniks). Aliquots (10 µL) of the samples dissolved in methanol were analyzed by flow injection analysis (FIA) using an isocratic gradient (50 A:50 B) of 0.1% formic acid in water (A) and 0.1% of formic acid in acetonitrile (B), at a flow rate of 10 µLmin^−1^ over 15 min. The mass spectrometer was operated in the ESI positive ion mode and in the high-resolution mode. The following optimized parameters were used: Capillary voltage: +4.5 kV; end plate offset: –500 V; nebulizer: 40 psi; dry gas: 4.0 L·min^−1^; dry heater: 200 °C; *m*/*z* 100–2000; acquisition mode: full scan acquisition with a scan rate of 1.0 Hz. Prior to analysis, the mass scale was calibrated by infusion of ESI low concentration tune mix at a 15 µL·min^−1^ flow rate. Recalibration of acquired data was performed via lock mass internal calibration (1221.9906) located within the source. Data processing was performed using the Data Analysis 4.1 software.

### 3.1. X-Ray Crystallographic Analysis

X-ray crystallographic data for compounds **4a**, **7b**, and **7d** were collected from single crystals using an area detector diffractometer (Bruker AXS-KAPPA APEX II, Madison, WI, USA) at room temperature and graphite-monochromated Mo Kα (λ = 0.71073 Å) radiation. Cell parameters were retrieved using Bruker SMART software and refined with Bruker SAINT [46] on all observed reflections. Absorption corrections were applied using SADABS [47]. The structures were solved by direct methods using SHELXT [48] and refined with full-matrix least-squares refinement against F^2^ using SHELXL [49]. All the programs are included in the WINGX package (version 2014.01) [50]. All non-hydrogen atoms were refined anisotropically, and the hydrogen atoms were inserted in idealized positions, riding on the parent C atom, except for those connected to nitrogen atoms, placed according to the electron density maps. Drawings were made using Mercury CSD 3.9 (Build RC1) [51]. Geometrical parameters such as angles between planes and torsion angles were calculated using Olex^2^ − 1.2.9 [52], while intermolecular interactions were determined using Platon 30715 [53] and visualized using Mercury. Despite several attempts, good quality crystals of compound **7d** could not be obtained, a circumstance that is reflected in the refinement of the crystal structure. Relevant details of the X-ray data analysis are displayed in Table 6 (*cf.* X-ray diffraction studies section). 

Crystallographic data for compounds **4a**, **7b**, and **7d** were deposited with the Cambridge Crystallographic Data Centre (CCDC 1496341, 1911742, and 1911743) and can be obtained free of charge from: CCDC, 12 Union Road, Cambridge CB2 1EZ, UK (Fax: 44-1223-226033; e-mail: deposit@ccdc.cam.ac.uk) or http://www.ccdc.cam.ac.uk/deposit.

### 3.2. General Procedure for the Preparation of DHPMs ***4a**,**b***

The DHPMs **4a**,**b** were prepared as previously described [31]. Briefly, a mixture of aldehyde (1 mmol), ethyl benzoylacetate (1 mmol), thiourea (1 mmol), and quartz (0.5 g) in ethanol (15 mL) was refluxed for 3 h. After completion of the reaction, as monitored by thin layer chromatography (tlc), the catalyst was filtered off and the solution was kept overnight. The precipitate formed on setting was filtered under suction and then recrystallized from ethanol to afford the pure product **4a**,**b**.

#### 3.2.1. Ethyl 4,6-diphenyl-2-thioxo-1,2,3,4-tetrahydropyrimidine-5-carboxylate (**4a**)

Yield 85%; *R*_f_ 0.54 (tlc, silica, 2:1 hexane:ethyl acetate); mp 193–195 °C [lit [54], 192 °C]; ^1^H-NMR (400 MHz, DMSO-*d*_6_) δ 0.73 (t, 3H, *J* 7.0, C**H_3_**CH_2_O), 3.74 (q, 2H, *J* 7.1, CH_3_C**H_2_**O), 5.27 (d, 1H, *J* 3.4, C4-**H**), 7.30–7.41 (m, 10 H, Ph-**H**), 9.75 (s, 1H, exchangeable, N3-**H**), 10.47 (s, 1H, exchangeable, N1-**H**); ^13^C-NMR (400 MHz, DMSO-*d*_6_) δ 13.31 (**C**H_3_), 54.08 (**C4**), 59.44 (**C**H_2_), 101.82 (**C5**), 126.38, 127.68, 127.78, 128.63, 128.66, 129.08 (Ph-**C**H), 133.98, 143.00 (Ph-**C_quat_**), 145.79 (**C6**), 164.87 (**C**=O), 174.51 (**C**=S); ESI-MS *m*/*z* 339 [MH]^+^; ESI-MS/MS (339) *m*/*z* 322, 280, 276, 263, 234, 219; HRMS (ESI-TOF) Calcd for C_19_H_19_N_2_O_2_S, *m*/*z* 339.1162 ([MH]^+^), Found 339.1154 (Δ = –2.4 ppm).

#### 3.2.2. Ethyl 4-(4-methoxyphenyl)-6-phenyl-2-thioxo-1,2,3,4-tetrahydropyrimidine-5-carboxylate (**4b**)

Yield 68%; *R*_f_ 0.43 (tlc, silica, 2:1 hexane:ethyl acetate); mp 130–132 °C; ^1^H-NMR (400 MHz, DMSO-*d*_6_) δ 0.74 (t, 3H, *J* 7.0, C**H_3_**CH_2_O), 3.73 (q, 2H, *J* 7.0, CH_3_C**H_2_**O), 3.75 (s, 3H, OC**H_3_**), 5.22 (s, 1H, C4-**H**), 6.96 (d, 2H, *J_ortho_* 7.8, 4-OCH_3_Ph-C3,C5-**H**), 7.30 (m, 5H, Ph-**H**), 7.40 (d, 2H, *J_ortho_* 7.8, 4-OCH_3_Ph-C2,C6-**H**), 9.67 (s, 1H, exchangeable, N3-**H**), 10.40 (s, 1H, exchangeable, N1-**H**); ^13^C-NMR (400 MHz, DMSO-*d*_6_) δ 13.30 (**C**H_3_), 53.52 (**C4**), 55.08 (O**C**H_3_), 59.44 (**C**H_2_), 102.08 (**C5**), 114.0 (4-OCH_3_Ph-**C3**,**C5**), 127.60 (4-OCH_3_Ph-**C2**,**C6**), 127.63, 128.60, 129.08 (Ph-**C**H), 134.04 (Ph-**C1**), 135.16 (4-OCH_3_Ph-**C1**), 145.46 (**C6**), 158.82 (4-OCH_3_Ph-**C4**), 164.85 (**C**=O), 174.30 (**C**=S); ESI-MS *m*/*z* 369 [MH]^+^; ESI-MS/MS (369) *m*/*z* 352, 310, 293, 264, 249, 217; HRMS (ESI-TOF) Calcd for C_20_H_21_N_2_O_3_S, *m*/*z* 369.1273 ([MH]^+^), Found 369.1267 (Δ = −1.6 ppm).

#### 3.2.3. Synthesis of the Thiazolo[3,2-*a*]pyrimidine Bromides **7a–d** and the Neutralized Compound **9**

A mixture of each DHPM **4a**,**b** (1 mmol) and the appropriate phenacyl bromide derivative **5** (1 mmol) in ethanol was heated under reflux for 12 h. The reaction mixture was monitored by tlc. The solvent was removed under reduced pressure. The residue was washed several times with a mixture of *n*-hexane:ethyl acetate 2:1. After washing, the final precipitate was dried to give the bromide salt of thiazolo[3,2-*a*]pyrimidines **7a**–**d** with good yields. Compound **7b** was neutralized to the corresponding product **9** using a saturated sodium carbonate solution.

#### 3.2.4. 6-(Ethoxycarbonyl)-3,5,7-triphenyl-5*H*-thiazolo[3,2-*a*]pyrimidin-8-ium bromide (**7a**)

Yield 80%; *R*_f_ 0.44 (tlc, silica, 2:1 hexane:ethyl acetate); mp 205–207 °C; ^1^H-NMR (400 MHz, CDCl_3_) δ 0.78 (t, 3H, *J* 7.0, C**H_3_**CH_2_O), 3.85 (q, 2H, *J* 7.0, CH_3_C**H_2_**O), 6.44 (s, 1H, C5-**H**), 6.80 (d, 2H, *J* 7.5, Ph-C**H**), 7.14–7.55 (m, 14H, Ph-C**H** and C2-**H**), 13.40 (br, 1H, N^+^8-**H**); ^13^C-NMR (400 MHz, CDCl_3_) δ 13.43 (**C**H_3_CH_2_O), 59.81 (**C5**), 61.10 (CH_3_**C**H_2_O), 103.33 (**C6**), 113.72 (**C2**), 126.70 (Ph-CH), 127.00 (Ph-**C_quat._**), 128.35, 129.15, 129.40, 129.62, 129.91, 130.90, 130.95 (Ph-**C**H), 131.75, 138.92 (Ph-**C_quat._**), 139.80 (**C3**), 144.12 (**C7**), 160.93 (**C9**), 164.70 (O-**C**=O); ESI-MS/MS (439) *m*/*z* 393, 315, 263, 219; HRMS (ESI-TOF) Calcd for C_27_H_23_N_2_O_2_S^+^, 439.1480 [M]^+^, Found 439.1480 (Δ = 0.0 ppm).

#### 3.2.5. 6-(Ethoxycarbonyl)-3-(4-methoxyphenyl)-5,7-diphenyl-5H-thiazolo[3,2-*a*]pyrimidin-8-ium bromide (**7b**)

Yield 83%; *R*_f_ 0.55 (tlc, silica, 2:1 hexane:ethyl acetate); mp 236–238 °C; ^1^H-NMR (400 MHz, CDCl_3_) δ 0.78 (t, 3H, *J* 7.1, C**H_3_**CH_2_O), 3.85 (q, 2H, *J* 7.1, CH_3_C**H_2_**O), 3.87 (s, 3H, OC**H_3_**), 6.41 (s, 1H, C5-**H**), 6.85 (d, 2H, *J* 7.5, Ph-C**H**), 6.92 (d, 2H, *J* 8.5, 4-OCH_3_Ph-**C3**,**5**), 7.10 (d, 2H, *J* 8.5, 4-OCH_3_Ph-**C2**,**6**), 7.15 (s, 1H, C2-**H**), 7.17–7.44 (m, 6H, Ph-C**H**), 7.53 (d, 2H, *J* 7.7, Ph-C**H**), 13.34 (br, 1H, N^+^8-**H**); ^13^C-NMR (400 MHz, CDCl_3_) δ 13.44 (**C**H_3_CH_2_O), 55.63 (O**C**H_3_), 59.63 (**C5**), 61.10 (CH_3_**C**H_2_O), 103.10 (**C6**), 113.00 (**C2**), 114.55 (Ph-**C**H), 118.91 (4-OCH_3_Ph**-C1**), 126.64, 128.35, 129.17, 129.38, 129.60, 130.91, 131.38 (Ph-**C**H), 131.71, 139.00 (Ph-**C_quat._**), 139.84 (**C3**), 144.24 (**C7**), 160.70 (**C9**), 161.56 (**C**-OCH_3_), 164.74 (O-**C**=O); ESI-MS *m*/*z* 469 [M]^+^, ESI-MS/MS (469) *m*/*z* 423, 345, 263, 219, 191; HRMS (ESI-TOF) Calcd for C_28_H_25_N_2_O_3_S^+^, 469.1586 [M]^+^, Found 469.1598 (Δ = 2.6 ppm).

#### 3.2.6. 6-(Ethoxycarbonyl)-3-(4-fluorophenyl)-5,7-diphenyl-5H-thiazolo[3,2-*a*]pyrimidin-8-ium bromide (**7c**)

Yield 74%; *R*_f_ 0.52 (tlc, silica, 2:1 hexane:ethyl acetate); mp 208–210 °C; ^1^H-NMR (400 MHz, CDCl_3_) δ 0.78 (t, 3H, *J* 7.1, C**H_3_**CH_2_O), 3.85 (m, 2H, CH_3_C**H_2_**O), 3.87 (s, 3H, OC**H_3_**), 6.36 (s, 1H, C5-**H**), 6.83 (d, 2H, *J* 7.5, Ph-C**H**), 7.10–7.27 (m, 7H, Ph-C**H**), 7.30 (s, 1H, C2-**H**), 7.37-7.43 (m, 3H, Ph-C**H**), 7.52 (d, 2H, *J* 7.1, Ph-C**H**), 13.30 (br, 1H, N^+^8-**H**); ^13^C-NMR (400 MHz, CDCl_3_) δ 13.43 (**C**H_3_CH_2_O), 59.93 (**C5**), 61.13 (CH_3_**C**H_2_O), 103.36 (**C6**), 114.60 (**C2**), 116.30, 116.50 (4-FPh-**C**H), 123.10 (4-FPh-**C1**), 126.66, 128.34, 129.23, 129.32, 129.70, 130.91, 130.85 (Ph-**C**H), 131.75 (Ph-**C_quat._**), 132.13, 132.22 (4-FPh-**C**H), 138.51 (**C3**), 138.95 (Ph-**C_quat._**), 143.83 (**C7**), 160.82 (**C9**), 164.60 (O-**C**=O), 162.86, 165.40 (4-FPh-**C**4); ESI-MS *m*/*z* 457 [M]^+^, ESI-MS/MS (457) *m*/*z* 411, 333, 263, 219, 191; HRMS (ESI-TOF) Calcd for C_27_H_22_FN_2_O_2_S^+^, 457.1386 [M]^+^, Found 457.1396 (Δ = 2.2ppm).

#### 3.2.7. 6-(Ethoxycarbonyl)-5-(4-methoxyphenyl)-3,7-diphenyl-5*H*-thiazolo[3,2-*a*]pyrimidin-8-ium bromide (**7d**)

Yield 70%; *R*_f_ 0.40 (tlc, silica, 2:1 hexane:ethyl acetate); mp 193-195 ^o^C; ^1^H-NMR (400 MHz, CDCl_3_) δ 0.78 (t, 3H, *J* 7.1, C**H_3_**CH_2_O), 3.74 (s, 3H, OC**H_3_**), 3.85 (q, 2H, *J* 7.1, CH_3_C**H_2_**O), 6.73 (s, 1H, C5-**H**), 6.65 (d, 2H, *J* 8.6, Ph-C**H**), 6.71 (d, 2H, *J* 8.6, 4-OCH_3_Ph-**C3**,**5**), 7.17 (d, 3H, Ph-C**H** and C2-**H**), 7.41–7.47 (m, 5H, Ph-C**H**), 7.54 (d, 2H, *J* 7.0, Ph-C**H**), 13.37 (br, 1H, N^+^8-**H**); ^13^C-NMR (400 MHz, CDCl_3_) δ 13.43 (**C**H_3_CH_2_O), 55.42 (O**C**H_3_), 59.41 (**C5**), 61.06 (CH_3_**C**H_2_O), 103.45 (**C6**), 113.26 (**C2**), 114.41 (Ph-**C**H), 127.15 (Ph**-C_quat._**), 128.24, 128.37, 129.18, 129.36, 129.91, 130.87, 130.96 (Ph-**C**H), 131.71, 131.77 (Ph-**C_quat._**), 139.88 (**C3**), 143.80 (**C7**), 160.50 (**C**-OCH_3_), 160.65 (**C9**), 164.74 (O-**C**=O); ESI-MS *m*/*z* 469 [M]^+^, ESI-MS/MS (469) *m*/*z* 423, 315, 293, 263, 249; HRMS (ESI-TOF) Calcd for C_28_H_25_N_2_O_3_S^+^, 469.1586 [M]^+^, Found 469.1595 (Δ = 1.9 ppm).

#### 3.2.8. Ethyl 3-(4-methoxyphenyl)-5,7-diphenyl-5*H*-thiazolo[3,2-*a*]pyrimidine-6-carboxylate (**9**)

Yield 70%; *R*_f_ 0.52 (tlc, silica, 2:1 hexane:ethyl acetate); mp 205-207 ^o^C; ^1^H-NMR (400 MHz, CDCl_3_) δ 0.78 (t, 3H, *J* 7.1, C**H_3_**CH_2_O), 3.82 (q, 2H, *J* 7.1, CH_3_C**H_2_**O), 3.86 (s, 3H, OC**H_3_**), 6.18 (s, 1H, C2-**H**), 6.27 (s, 1H, C5-**H**), 6.91 (d, 2H, *J* 8.4, Ar-C**H**), 6.96 (d, 2H, *J* 7.0, Ar-C**H**), 7.08 (d, 2H, *J* 8.4, Ar-C**H**), 7.174–7.40 (m, 8H, Ar-C**H**); ^13^C-NMR (400 MHz, CDCl_3_) δ 13.62 (**C**H_3_CH_2_O), 55.53 (O**C**H_3_), 58.36 (**C5**), 59.75 (CH_3_**C**H_2_O), 100.57 (**C6**), 103.04 (**C2**), 114.21 (Ar-**C**H), 121.82 (**C3**), 126.56, 127.70, 128.26, 128.40, 128.50, 130.60 (Ph-**C**H), 139.70, 141.04, 142.40 (Ar-**C_quat._**), 156.26 (**C7**), 160.75 (**C**-OCH_3_), 166.53 (**C9**), 167.04 (O-**C**=O).

### 3.3. Molecular Docking Studies

The protein structure was prepared starting from the crystallographic complex of human AChE in complex with the AChE inhibitor Territrem B (PDB ID: 4m0f, chain A). Territrem B and other non-protein molecules were deleted and only the enzyme chain was retained. Hydrogen atoms and missing heavy atoms were added. Zero occupancy side chains, polar hydrogen atoms, and the positions of asparagine and glutamine side chain amidic groups were optimized and assigned the lowest energy conformation. The Autodock Vina software [39] was used for the molecular docking process using a flexible protein-flexible ligand approach. The protein flexible regions were defined as spherical regions with a radius of 11 Å centered at the geometrical center of the Territrem B binding site. The docking search space was defined as a cubic 30 × 45 × 30 Å^3^ box co-centrical with the flexible region. Docking was performed with an exhaustiveness of 32 and the top 100 binding modes within 10 kcal/mol of the top binding mode were recorded and analyzed. Protein-ligand analyses were performed with UCSF Chimera [55].

### 3.4. Enzyme Inhibition Activity

#### 3.4.1. Enzyme and Assay Reagents

Recombinant Human Acetylcholinesterase (r*h*AChE, catalog number 7574-CE, expressed in Chinese hamster ovary cells) was purchased from R&D Systems. 5,5′-Dithiobis(2-nitrobenzoic acid) (DTNB, Ellman’s reagent), acetylthiocholine iodide (ACTI), and the methyl paraoxon used as reference AChE inhibitor were purchased from Sigma-Aldrich (Schnelldorf, Germany).

#### 3.4.2. *In Vitro* Acetylcholinesterase Activity Assay

The inhibitory activities of the synthesized compounds on *h*AChE were evaluated using a colorimetric Ellman-based method, with methyl paraoxon as the reference compound [43]. The inhibitors were dissolved in DMSO, acetylthiocholine iodide (ACTI, 20 mM) was dissolved in distilled water, and the dithiobis(2-nitrobenzoic acid) (DTNB, 10 mM) and methyl paraoxon (20 mM) were dissolved in assay buffer. Prior to use, all refrigerated solutions were allowed to reach room temperature. Enzyme (100 µL, 0.2 µg/mL) and inhibitor solutions (20 µL) were added into a cuvette containing 4.0 mL assay buffer (100 mM sodium phosphate, pH 7.4/1 mM EDTA). After 10 min incubation at room temperature, required aliquots of the DTNB (20 µL) and the ACTI (20 µL) solutions were added. After gently mixing, the total mixture was incubated at 37 °C for 35 min. The absorption was measured at 405 nm on a Shimadzu 3101PC UV-vis spectrophotometer and corrected for background. The specific inhibition extents were calculated in reference to measurements performed under the same conditions in the absence of inhibitor and expressed as percentage inhibition. For comparative purposes, the standard inhibitor, methyl paraoxon, was tested concomitantly, giving >95–100% inhibition in all instances. The enzyme activity was determined in the presence of concentrations up to 50 µM for any given inhibitor. The same concentrations were used for methyl paraoxon. The measurements were conducted immediately after sample preparation. Inhibition curves were produced to calculate the IC_50_ values.

## 4. Conclusions

In summary, we synthesized a series of fused thiazolo[3,2-*a*]pyrimidines in good yields from 3,4-dihydropyrimidinethione precursors. All compounds were fully characterized by 1D- and 2D-NMR, high resolution ESI-MS/MS, and single crystal X-ray diffraction analysis, which confirmed a regioselective 5*H* cyclization of the DHPMs. All target compounds were screened *in vitro* as *h*AChE inhibitors via an Ellman-based colorimetric assay and showed good inhibition activities (better than 70%) at 10 µM, with IC_50_s in the 1 μM range. Molecular docking simulations for all target products into *h*AChE were performed and confirmed strong binding to the peripheral anionic site of the enzyme, at the mouth of the catalytic gorge, which funnels access to the catalytic site. These preliminary data set the ground for further improvement of the core structure and provide a new and promising starting point to develop new acetylcholinesterase inhibitors and explore novel treatment options against Alzheimer’s disease.

## Figures and Tables

**Table 1:** ^1^H- and ^13^C-NMR chemical shifts (δ, in ppm) for the methine proton (C5-H) and carbon (C5) for all compounds discussed in the paper.

Comp.	4a	4b	7a	7b	7c	7d	9
^1^H-NMR	5.27	5.22	6.44	6.41	6.36	6.73	6.27
^13^C-NMR	54.08	53.52	59.87	59.63	59.93	59.41	58.36

**Table 2:** Comparison of the relevant carbon chemical shifts (δ, in ppm) for the protonated compound **7b** and the non-protonated compound **9.**

Comp.	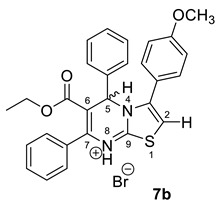	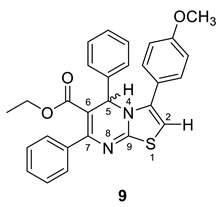
C2	113.00	103.04
C5	59.63	58.36
C6	103.10	100.57
C7	144.24	156.26
C9	160.70	166.53
C=O	164.74	167.04

**Table 3:** Selected geometrical parameters for compound **4a.**

**Bond**	**Bond Length [Å]**	**Angle**	**Angle [°]**
N(1)-C(2)	1.360(3)	C(2)-N(1)-C(6)	123.1(2)
N(1)-C(6)	1.387(3)	N(3)-C(2)-N(1)	116.4(2)
S(2)-C(2)	1.680(3)	N(3)-C(2)-S(2)	121.9(2)
N(3)-C(2)	1.327(3)	N(1)-C(2)-S(2)	121.70(18)
N(3)-C(4)	1.469(3)	C(2)-N(3)-C(4)	126.8(2)
C(4)-C(5)	1.508(3)	N(3)-C(4)-C(5)	108.95(18)
C(5)-C(6)	1.353(3)	N(3)-C(4)-C(20)	110.4(2)
**Torsion Angle (R-molecule)**	**Angle [°]**	C(5)-C(4)-C(20)	113.3(2)
O(7)-C(7)-C(5)-C(4)	−11.5(4)	C(6)-C(5)-C(7)	125.1(2)
		C(6)-C(5)-C(4)	120.6(2)
		C(7)-C(5)-C(4)	114.2(2)
		C(5)-C(6)-C(30)	126.6(2)
		N(1)-C(6)-C(30)	113.9(2)
		C(5)-C(6)-N(1)	119.5(2)
**Angles between Planes**		**Angles [°]**	
N1-C2-N3- C4-C5-C6	C20-…-C25	85.68(13)	
N1-C2-N3- C4-C5-C6	C30-…-C35	66.15(17)	

**Table 4:** Selected geometrical parameters for compounds **7b** and **7d.**

Molecule	7b	7d
**Bond Length [Å]**		
S(1)-C(2)	1.731(4)	1.717(4)
S(1)-C(9)	1.712(3)	1.706(4)
C(3)-C(2)	1.335(5)	1.339(6)
N(4)-C(3)	1.425(4)	1.410(5)
N(4)-C(5)	1.503(4)	1.488(5)
C(5)-C(6)	1.520(5)	1.514(5)
C(6)-C(7)	1.339(5)	1.340(5)
N(8)-C(7)	1.417(4)	1.394(5)
N(8)-C(9)	1.340(4)	1.335(5)
N(4)-C(9)	1.337(4)	1.332(4)
**Angles [°]**		
N(4)-C(3)-C(ring)	122.3(3)	122.7(4)
C(2)-C(3)-C(ring)	126.8(3)	126.2(4)
C(3)-N(4)-C(5)	123.5(3)	123.9(3)
C(9)-N(4)-C(3)	113.2(3)	112.6(3)
C(9)-N(4)-C(5)	121.7(3)	123.5(3)
N(4)-C(5)-C(6)	108.7(3)	109.3(3)
N(4)-C(5)-C(ring)	108.7(3)	111.2(3)
C(6)-C(5)-C(ring)	114.0(3)	110.6(3)
C(7)-C(6)-C(10)	126.0(3)	122.2(3)
C(7)-C(6)-C(5)	123.4(3)	124.0(3)
C(10)-C(6)-C(5)	110.6(3)	113.7(3)
C(6)-C(7)-N(8)	119.1(3)	119.6(3)
C(6)-C(7)-C(ring)	129.7(3)	127.1(3)
N(8)-C(7)-C(ring)	111.1(3)	113.3(3)
C(9)-N(8)-C(7)	120.5(3)	120.8(3)
N(8)-C(9)-N(4)	122.3(3)	122.7(3)
N(8)-C(9)-S(1)	125.0(3)	123.9(3)
N(4)-C(9)-S(1)	112.7(3)	113.4(3)
**Torsion Angles [°]**		
O(10)-C(10)-C(6)-C(5)	21.9(6)	−32.7(6)
**Angles between planes [°]**		
Center ring ^a^–C5 phenyl ring ^b^	89.49(12)	83.33(11)
Center ring ^a^–C7 phenyl ring ^c^	127.57(12)	74.09(14)
Center ring ^a^–C3 phenyl ring ^d^	55.68(12)	56.66(12)

^a^—S1, C2, C3, N4, C5, C6, C7, N8, C9. ^b^—Phenyl ring bonded to C5. ^c^—Phenyl ring bonded to C7. ^d^—Phenyl ring bonded to C3.

**Table 5:** Interaction parameters for compounds **4a**, **7b**, and **7d.**

D-H^…^A	H^…^A [Å]	D^…^A [Å]	D-H^…^A [°]	Symmetry Operation
**Compound 4a**				
N(3)-H(3N)^...^O(7)	2.02	2.872(2)	159	½ − x,1/2 + y,1/2 − z
N(1)-H(1N)^...^S(2)	2.52	3.337(2)	173	−x,1 − y,1 − z
**Compound 7b**				
N8-H8^...^Br1^a^	2.48	3.199(3)	141	x,y,z
C32-H32^...^Br1^b^	2.81	3.742(4)	175	x, −1 + y, z
C18-H18^...^O10	2.49	3.397(6)	166	1 − x, 1/2 + y, 1/2 − z
C24-H24^...^O26	2.60	3.507(6)	166	1 − x, 1 − y, 1 − z
**Compound 7d**				
N8-H8^...^Br1^a^	2.28	3.167(3)	165	x, y, z
C19-H19^...^O10	2.51	3.372(5)	153	x, −1 + y, z
C21-H21C^...^O20	2.66	3.456(6)	140	2 − x, −y, 1 − z

^a^—classical hydrogen bond. ^b^—for reasons of clarity these contacts are not represented in Figure 5.

**Table 6:** Structure refinement data of compounds **4a**, **7b**, and **7d.**

Compound	4a	7b	7d
Absorption coefficient (mm^−1^)	0.194	1.694	1.680
F(000)	712	1128	1128
Crystal size (mm)	0.30 × 0.15 × 0.15	0.200 × 0.100 × 0.040	0.150 × 0.060 × 0.040
θ range (°)	2.776 to 26.497	2.808 to 26.358	2.873 to 26.440
Index ranges	–16 ≤ h ≤16, –10 ≤ k ≤ 10, −20 ≤ l ≤ 20	−18 ≤ h ≤18, −10 ≤ k ≤10, −19 ≤ l ≤ 26	−16 ≤ h ≤ 16, −8 ≤ k ≤ 8, −35 ≤ l ≤ 35
Reflections collected	49,517	14,223	99,174
Independent reflections	3618 [R(int) = 0.0299]	5233 [R(int) = 0.0695]	5395 [R(int) = 0.2244]
Completeness to Θ max (%)	98.4	99.6	99.9
Data/restraints/parameters	3618/1/219	5233/0/316	5395/1/320
Goodness-of-fit on F2	1.051	0.955	1.054
Final R indices [I > 2sigma(I)]	R1 = 0.0711. wR2 = 0.1922	R1 = 0.0491, wR2 = 0.1000	R1 = 0.0491, wR2 = 0.0873
R indices (all data)	R1 = 0.0842, wR2 = 0.2063	R1 = 0.1095, wR2 = 0.1210	R1 = 0.1299, wR2 = 0.1163
Extinction coefficient	n/a	n/a	n/a
Largest diff. peak/hole (e. Å^−3^)	0.872/−0.992	0.300 and −0.501	0.610 and −0.503

## Supplementary Materials

The supplementary materials are available online.
