# Supplementary material for: Synthesis, Crystal Structure, and Biological Evaluation of Fused Thiazolo[3,2-a]Pyrimidines as New Acetylcholinesterase Inhibitors
Source: Molecules. 2019 Jun 21;24(12):2306. doi: 10.3390/molecules24122306 (PMC6630738; doi:10.3390/molecules24122306)
Supplement: Supplementary file 1 [file molecules-24-02306-s001.pdf]

Synthesis, Crystal Structure and Biological Evaluation of Fused Thiazolo[3,2-*a*]pyrimidines as new Acetylcholinesterase Inhibitors

Mohamed Y. Mahgoub [1,2], **Awatef M. Elmaghraby** [1], **Abd-Elfttah A. Harb** [1], **João L. Ferreira da Silva** [2], **Gonçalo C. Justino** [2] and **M. Matilde Marques** [2]

1) Chemistry Department, Faculty of Science, South Valley University, Qena 83523, Egypt
2) Centro de Química Estrutural, Instituto Superior Técnico, Universidade de Lisboa, 1049-001 Lisboa, Portugal

Supplementary data:

Figure (S1). ^{1}H NMR spectrum (400 MHz, $CDCl_3$) of compound 7a

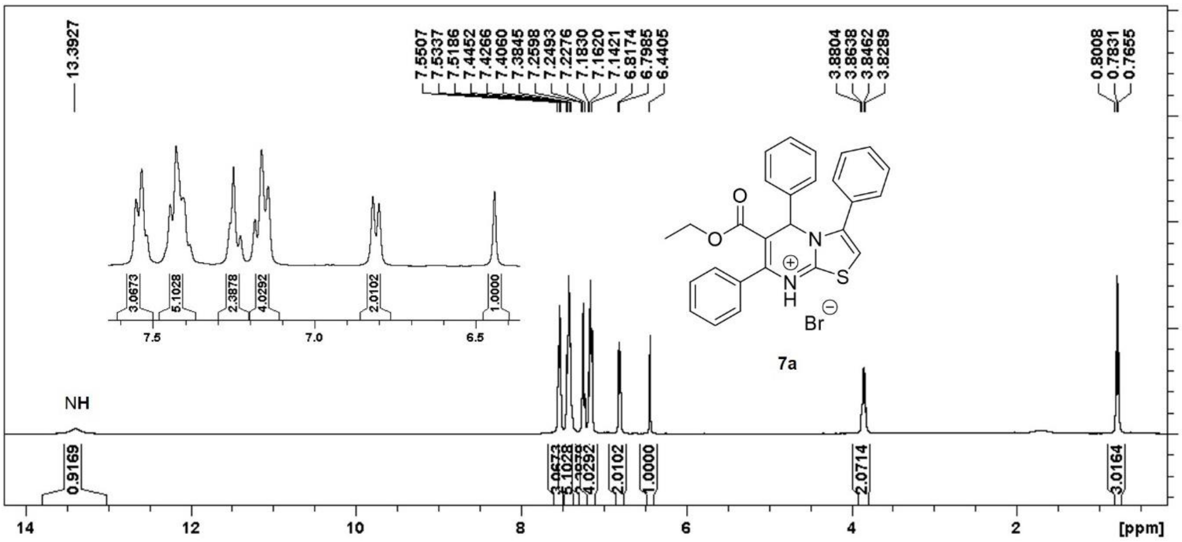

Figure (S2). ^{13}C NMR spectrum (400 MHz, $CDCl_3$) of compound 7a

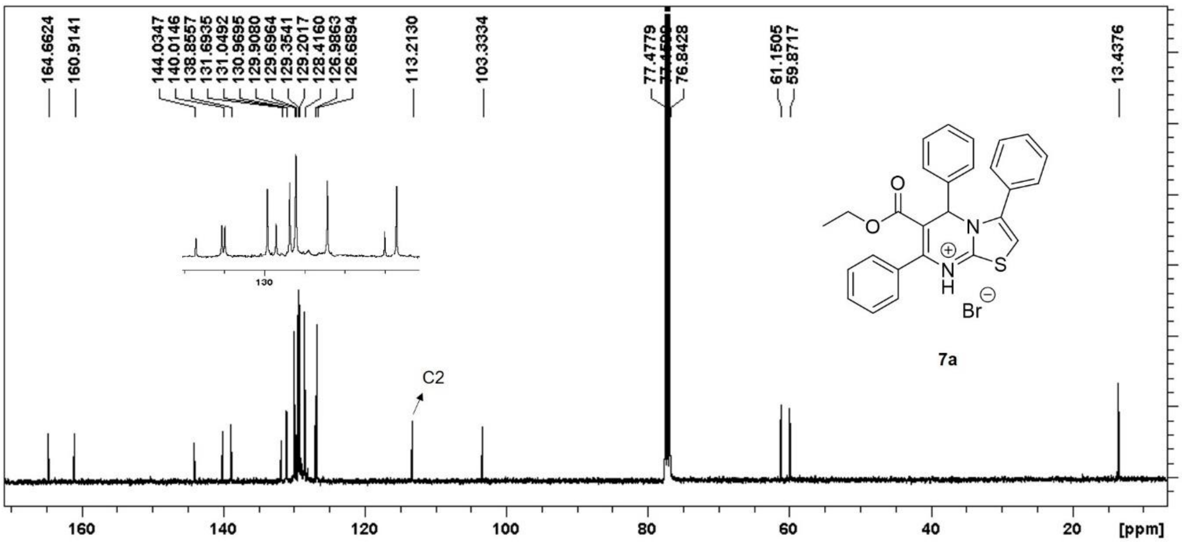

Figure (S3). Full scan HR-MS spectrum of compound 7a

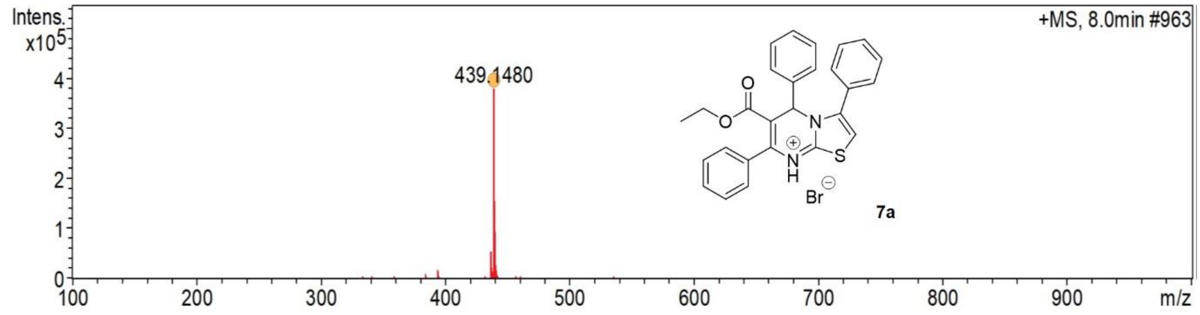

Figure (S4). ^{1}H NMR spectrum (400 MHz, $CDCl_3$) of compound 7b

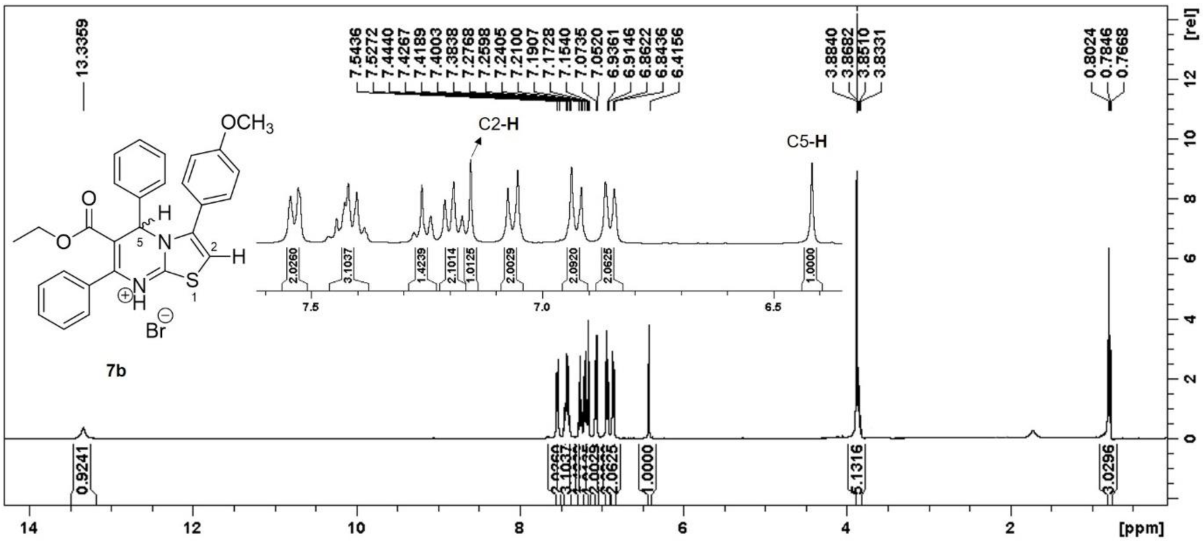

Figure (S5). ^{13}C NMR spectrum (400 MHz, $CDCl_3$) of compound 7b

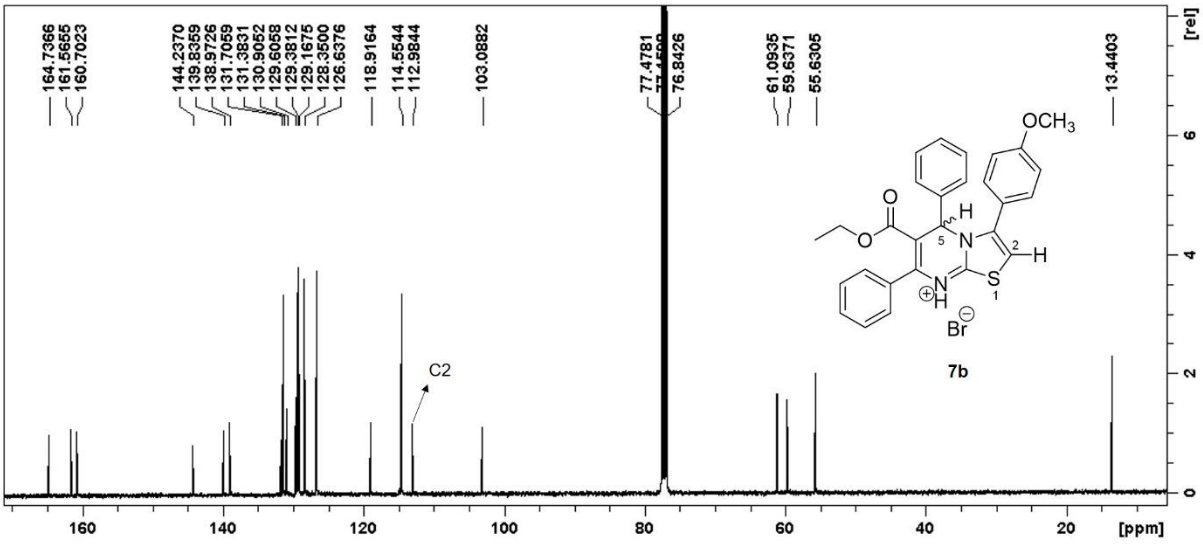

Figure (S6). Full scan HR-MS spectrum of compound 7b

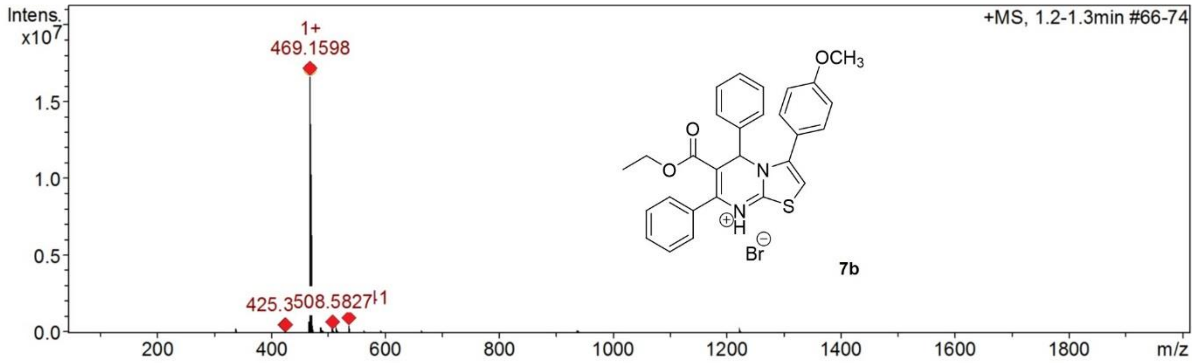

Figure (S7). ^{1}H NMR spectrum (400 MHz, $CDCl_3$) of compound 7c

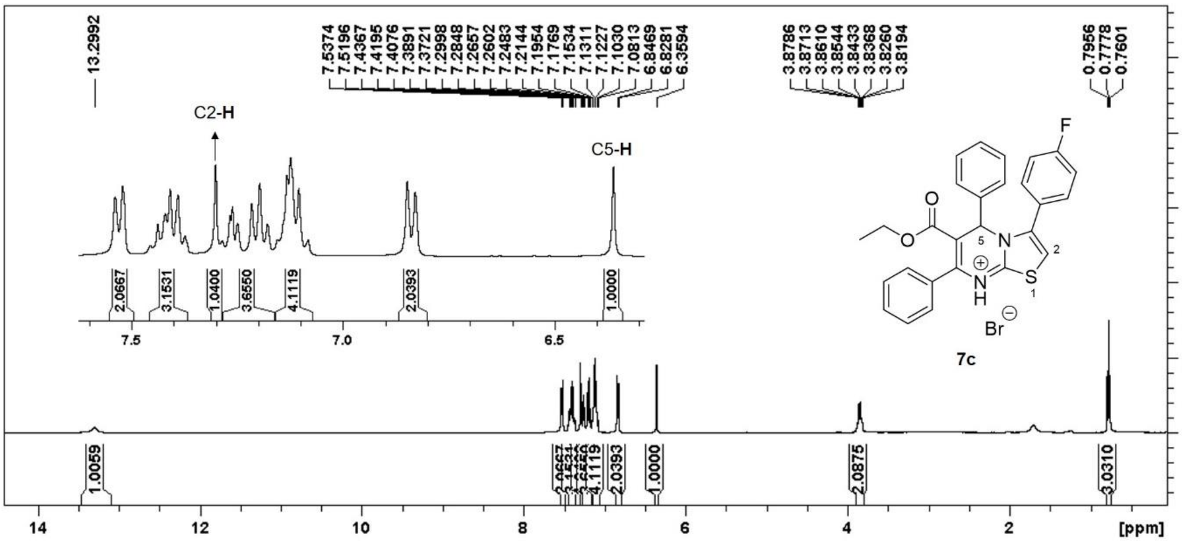

Figure (S8). ^{13}C NMR spectrum (400 MHz, $CDCl_3$) of compound 7c

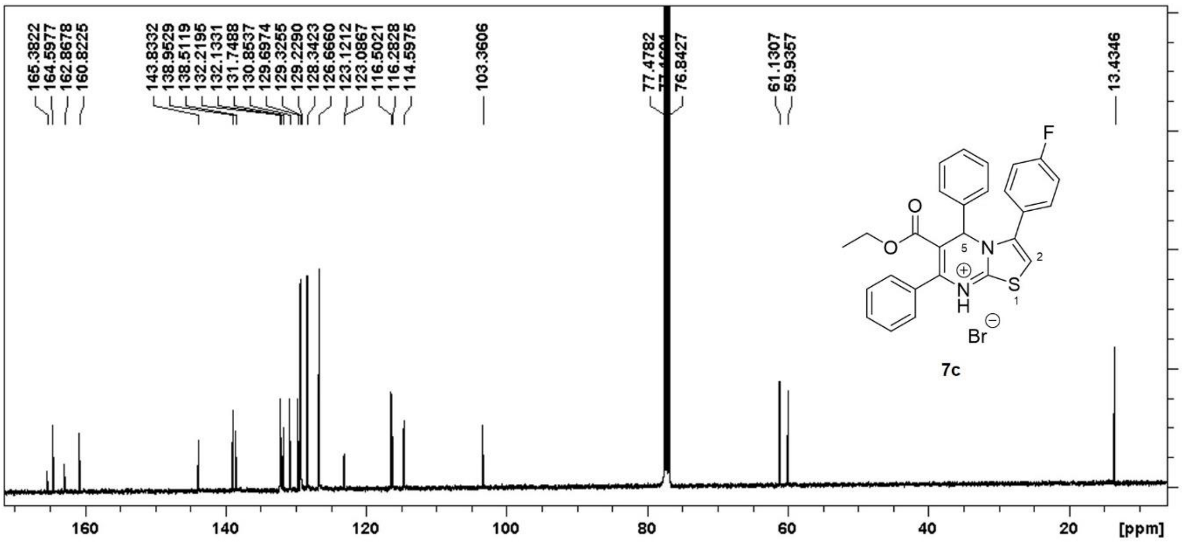

Figure (S9). Full scan HR-MS spectrum of compound 7c

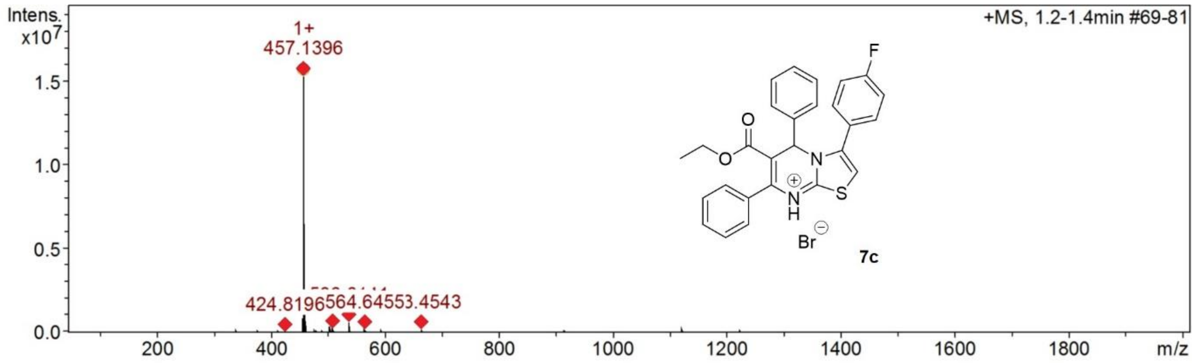

Figure (S10). ^1H NMR spectrum (400 MHz, $CDCl_3$) of compound 7d

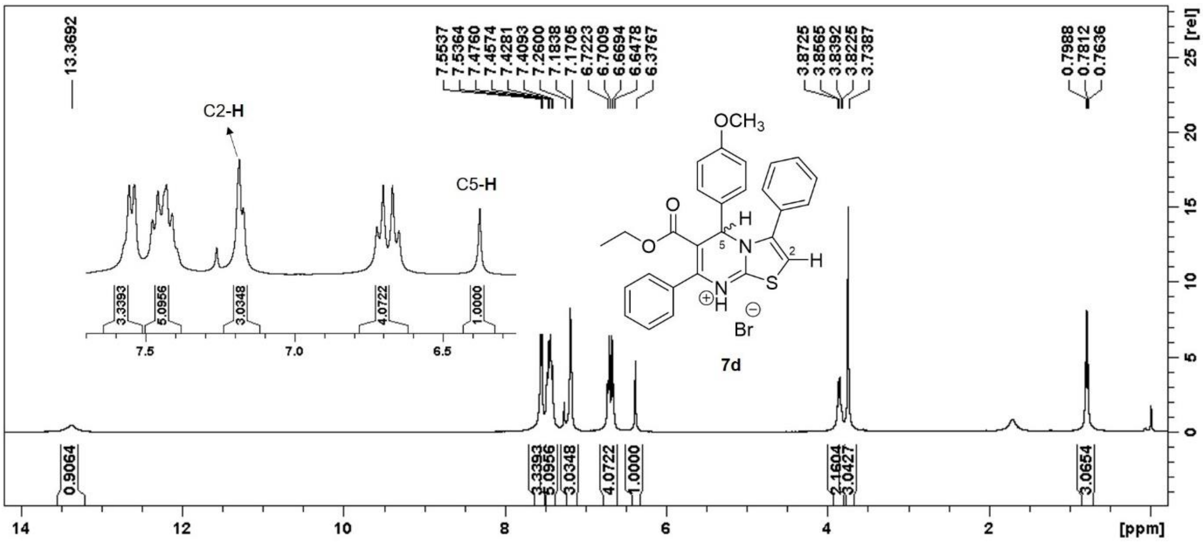

Figure (S11). ^{13}C NMR spectrum (400 MHz, $CDCl_3$) of compound 7d

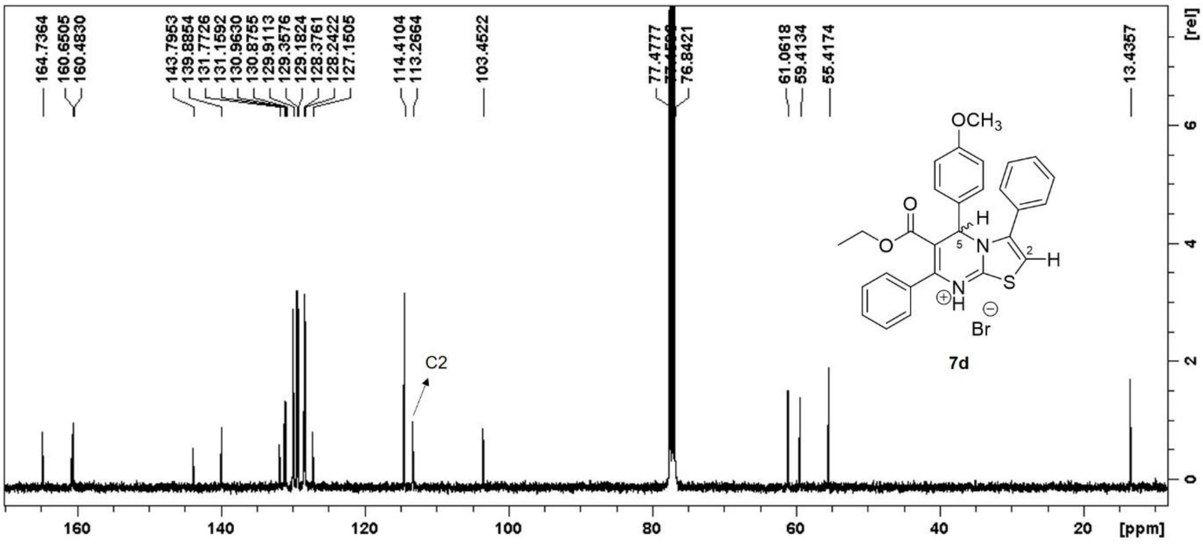

Figure (S12). Full scan ESI-MS spectrum of compound 7d

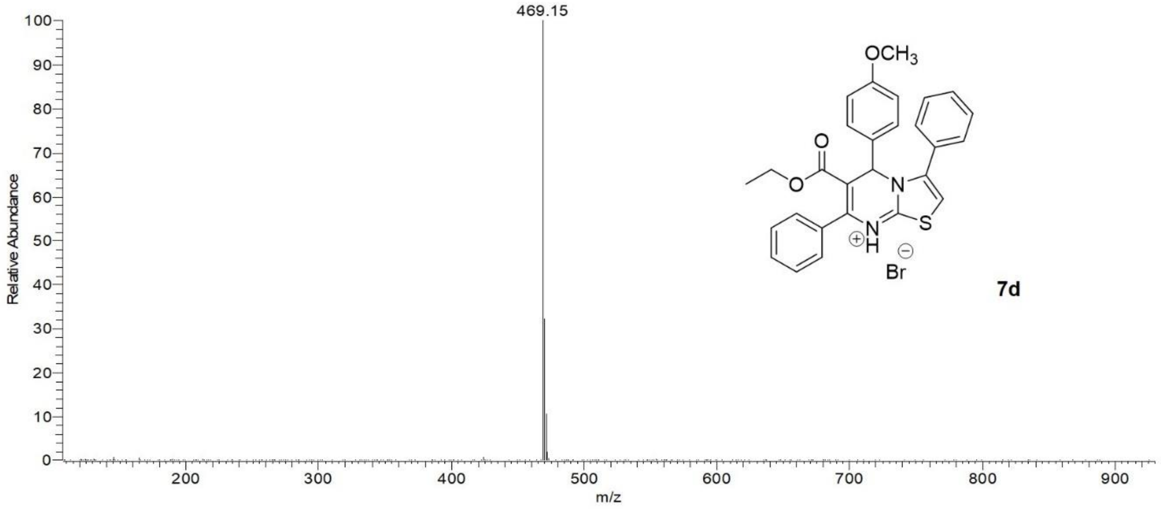

Figure (S13). ESI-MS/MS spectrum of compound 7d

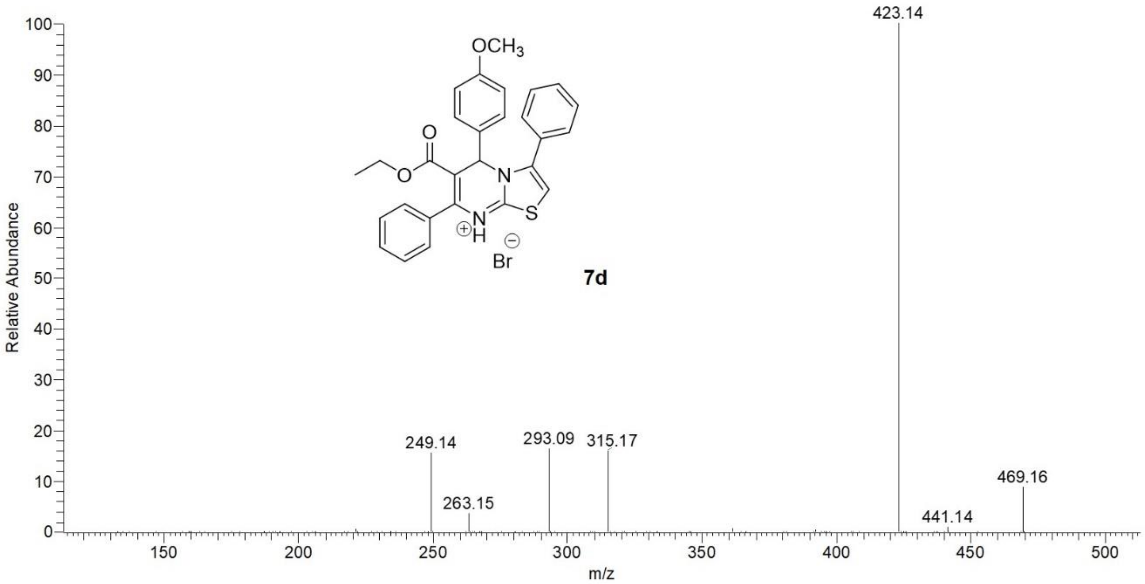

Figure (S14). ^{1}H NMR spectrum (400 MHz, $CDCl_3$) of compound 9

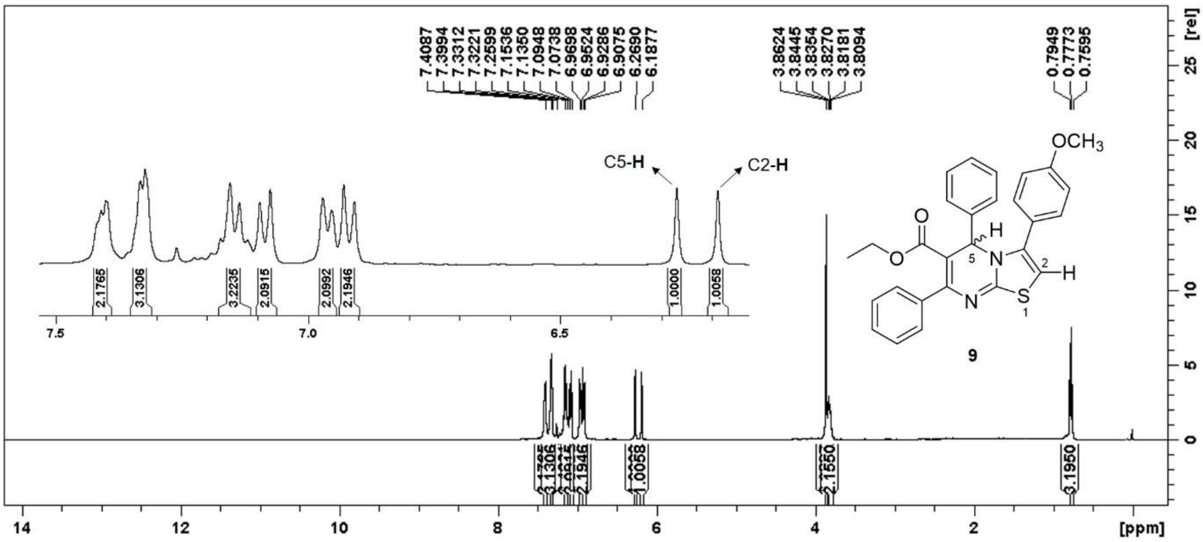

Figure (S15). ^{13}C NMR spectrum (400 MHz, $CDCl_3$) of compound 9

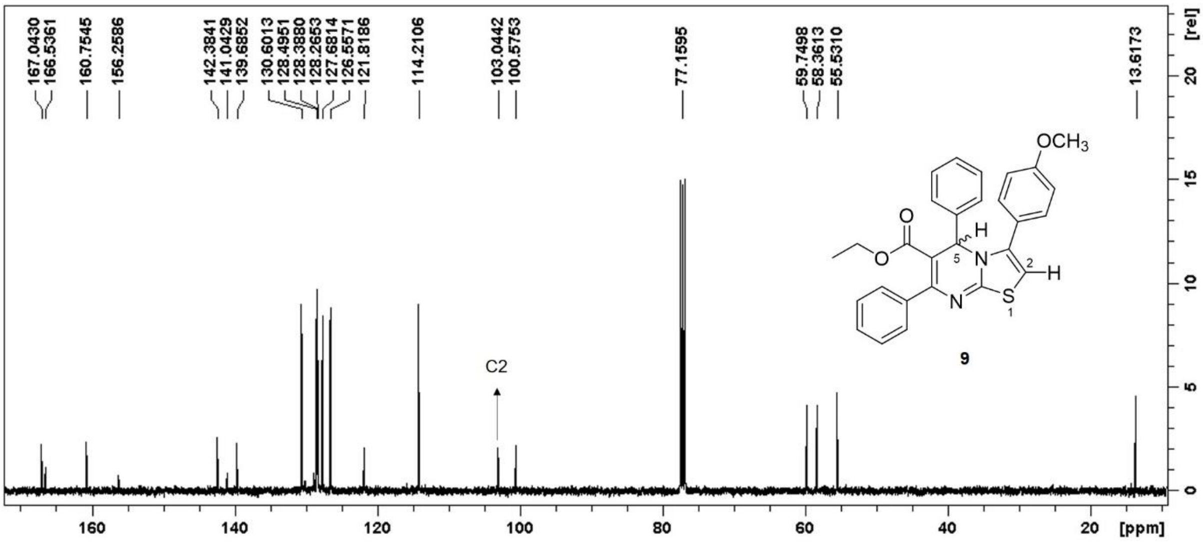

Table S1. Crystal parameters for compounds **4a, 7b,** and **7d**

Compound	4a	7b	7d
Empirical formula	$C_{19} H_{18} N_2 O_2 S$	$C_{56} H_{50} Br_2 N_4 O_6 S_2$	$C_{56} H_{50} Br_2 N_4 O_6 S_2$
Formula weight	338.41	1098.94	1098.94
Crystal system	Monoclinic	Monoclinic	Monoclinic
Space group	P 21/n	P 21/c	P 21/c
a (Å)<br>b (Å)<br>c (Å)	13.511(9).<br>8.255(6)<br>16.230(10)	14.814(4)<br>8.631(2)<br>20.986(6)	13.2324(8)<br>6.9831(4)<br>28.3593(18)
α (°)<br>β (°)<br>γ (°)	90<br>99.15(2)<br>90	90<br>104.370(13)<br>90	90<br>90.020(2)<br>90
Volume (Å^3)	1787(2)	2599.2(12)	2620.5(3)
Z	4	2	2
Packing index	64.2	65.7	64.8

Table S2. Geometrical parameters for compound **4a**

Bond	Bond length [Å]	Angle	Angle [°]
N(1)-C(2)	1.360(3)	C(2)-N(1)-C(6)	123.1(2)
N(1)-C(6)	1.387(3)	N(3)-C(2)-N(1)	116.4(2)
S(2)-C(2)	1.680(3)	N(3)-C(2)-S(2)	121.9(2)
N(3)-C(2)	1.327(3)	N(1)-C(2)-S(2)	121.70(18)
N(3)-C(4)	1.469(3)	C(2)-N(3)-C(4)	126.8(2)
C(4)-C(5)	1.508(3)	N(3)-C(4)-C(5)	108.95(18)
C(20)-C(4)	1.515(4)	N(3)-C(4)-C(20)	110.4(2)
C(5)-C(6)	1.353(3)	C(5)-C(4)-C(20)	113.3(2)
C(5)-C(7)	1.468(3)	C(6)-C(5)-C(7)	125.1(2)
C(6)-C(30)	1.492(3)	C(6)-C(5)-C(4)	120.6(2)
O(7)-C(7)	1.207(3)	C(7)-C(5)-C(4)	114.2(2)
C(7)-O(8)	1.325(3)	C(5)-C(6)-C(30)	126.6(2)
O(8)-C(9)	1.453(4)	N(1)-C(6)-C(30)	113.9(2)
C(9)-C(10)	1.334(7)	C(5)-C(6)-N(1)	119.5(2)
C(20)-C(21)	1.382(5)	O(7)-C(7)-O(8)	122.9(2)
C(21)-C(22)	1.389(5)	O(7)-C(7)-C(5)	123.0(2)
C(23)-C(22)	1.373(9)	O(8)-C(7)-C(5)	114.0(2)
C(23)-C(24)	1.358(9)	C(7)-O(8)-C(9)	117.9(3)
C(24)-C(25)	1.381(6)	C(10)-C(9)-O(8)	112.9(4)
C(25)-C(20)	1.392(4)	C(21)-C(20)-C(25)	119.4(3)
C(30)-C(31)	1.367(4)	C(21)-C(20)-C(4)	121.6(3)
C(31)-C(32)	1.394(5)	C(25)-C(20)-C(4)	119.1(3)

C(32)-C(33)	1.342(8)	C(20)-C(21)-C(22)	119.9(4)
C(33)-C(34)	1.368(8)	C(23)-C(22)-C(21)	120.1(5)
C(34)-C(35)	1.399(6)	C(24)-C(23)-C(22)	120.1(4)
C(30)-C(35)	1.378(5)	C(23)-C(24)-C(25)	120.9(5)
Torsion angle (R- molecule)	Angle [°]	C(24)-C(25)-C(20)	119.6(5)
O(7)-C(7)-C(5)-C(4)	-11.5(4)	C(31)-C(30)-C(35)	119.5(3)
		C(31)-C(30)-C(6)	121.1(3)
		C(35)-C(30)-C(6)	119.4(3)
		C(30)-C(31)-C(32)	119.7(4)
		C(33)-C(32)-C(31)	120.8(4)
		C(32)-C(33)-C(34)	120.4(4)
		C(33)-C(34)-C(35)	119.5(4)
		C(30)-C(35)-C(34)	120.0(4)
		C(2)-N(1)-C(6)	123.1(2)
		N(3)-C(2)-N(1)	116.4(2)
		N(3)-C(2)-S(2)	121.9(2)
		N(1)-C(2)-S(2)	121.70(18)

Angles between planes		Angles [°]
N1-C2-N3- C4-C5-C6	C20-…-C25	85.68(13)
N1-C2-N3- C4-C5-C6	C30-…-C35	66.15(17)

Table S3. Geometrical parameters for compounds**7b** and **7d**

Molecule	7b	7d
Bond length [Å]		
S(1)-C(9)	1.712(3)	1.706(4)
S(1)-C(2)	1.731(4)	1.717(4)
N(4)-C(9)	1.337(4)	1.332(4)
N(4)-C(3)	1.425(4)	1.410(5)
N(4)-C(5)	1.503(4)	1.488(5)
N(8)-C(9)	1.340(4)	1.335(5)
N(8)-C(7)	1.417(4)	1.394(5)
O(10)-C(10)	1.203(4)	1.203(5)
O(11)-C(10)	1.338(4)	1.319(5)
O(11)-C(12)	1.459(4)	1.448(5)
O(26)-C(23)	1.378(5)	
O(26)-C(27)	1.422(6)	
O(20)-C(17)		1.383(5)
O(20)-C(21)		1.414(6)
C(3)-C(2)	1.335(5)	1.339(6)
C(5)-C(6)	1.520(5)	1.514(5)
C(7)-C(6)	1.339(5)	1.340(5)
C(3)-C(20)	1.466(5)	
C(22)-C(3)		1.474(5)
C(5)-C(14)	1.522(5)	1.525(5)
C(6)-C(10)	1.494(5)	1.475(5)
C(7)-C(28)	1.497(5)	1.485(5)
C(12)-C(13)	1.504(6)	1.473(7)
C(14)-C(15)	1.387(5)	1.390(5)
C(15)-C(16)	1.384(6)	1.371(6)
C(17)-C(16)	1.373(7)	1.376(6)
C(18)-C(17)	1.383(7)	1.373(6)
C(19)-C(18)	1.396(6)	1.392(5)
C(14)-C(19)	1.384(5)	1.356(5)
C(20)-C(21)	1.385(5)	
C(21)-C(22)	1.386(5)	
C(22)-C(23)	1.377(6)	1.378(6)
C(23)-C(24)	1.395(6)	1.389(6)
C(24)-C(25)	1.371(5)	1.374(8)
C(20)-C(25)	1.397(5)	
C(26)-C(25)		1.358(8)
C(27)-C(26)		1.389(7)
C(22)-C(27)		1.393(6)
C(28)-C(29)	1.390(5)	1.365(6)
C(29)-C(30)	1.396(5)	1.378(6)
C(30)-C(31)	1.378(6)	1.345(8)
C(31)-C(32)	1.381(6)	1.351(8)

C(33)-C(32)	1.371(5)	1.390(7)
C(28)-C(33)	1.395(5)	1.371(6)
Angles [°]		
C(9)-S(1)-C(2)	89.57(18)	89.3(2)
C(9)-N(4)-C(3)	113.2(3)	112.6(3)
C(9)-N(4)-C(5)	121.7(3)	123.5(3)
C(3)-N(4)-C(5)	121.7(3)	123.9(3)
C(9)-N(8)-C(7)	120.5(3)	120.8(3)
C(10)-O(11)-C(12)	115.8(3)	116.4(4)
C(23)-O(26)-C(27)	119.0(4)	
C(17)-O(20)-C(21)		117.9(4)
C(3)-C(2)-S(1)	113.7(3)	113.5(3)
C(2)-C(3)-N(4)	110.7(3)	111.2(3)
C(2)-C(3)-C(20)	126.8(3)	
N(4)-C(3)-C(20)	122.3(3)	
C(2)-C(3)-C(22)		126.2(4)
N(4)-C(3)-C(22)		122.7(4)
N(4)-C(9)-N(8)	122.3(3)	122.7(3)
N(4)-C(9)-S(1)	112.7(3)	113.4(3)
N(8)-C(9)-S(1)	125.0(3)	123.9(3)
N(4)-C(5)-C(6)	108.7(3)	109.3(3)
N(4)-C(5)-C(14)	108.7(3)	111.2(3)
C(6)-C(5)-C(14)	114.0(3)	110.6(3)
C(7)-C(6)-C(10)	126.0(3)	122.2(3)
C(7)-C(6)-C(5)	123.4(3)	124.0(3)
C(10)-C(6)-C(5)	110.6(3)	113.7(3)
C(6)-C(7)-N(8)	119.1(3)	119.6(3)
C(6)-C(7)-C(28)	129.7(3)	127.1(3)
N(8)-C(7)-C(28)	111.1(3)	113.3(3)
O(10)-C(10)-O(11)	123.5(3)	123.6(4)
O(10)-C(10)-C(6)	122.6(3)	123.7(4)
O(11)-C(10)-C(6)	113.9(3)	112.7(4)
O(11)-C(12)-C(13)	106.4(3)	107.6(4)
C(19)-C(14)-C(15)	119.5(4)	118.7(4)
C(19)-C(14)-C(5)	121.1(4)	121.7(3)
C(15)-C(14)-C(5)	119.4(3)	119.5(4)
C(16)-C(15)-C(14)	119.9(5)	120.2(4)
C(15)-C(16)-C(17)	120.4(5)	120.5(4)
C(18)-C(17)-C(16)	120.7(5)	120.0(4)
C(18)-C(17)-O(20)		124.5(4)
C(16)-C(17)-O(20)		115.5(4)
C(17)-C(18)-C(19)	118.8(5)	118.7(4)
C(14)-C(19)-C(18)	120.7(5)	121.8(4)
C(21)-C(20)-C(25)	117.0(4)	
C(21)-C(20)-C(3)	120.7(4)	
C(25)-C(20)-C(3)	122.2(3)	
C(20)-C(21)-C(22)	122.8(4)	

C(23)-C(22)-C(21)	119.0(4)	
C(23)-C(22)-C(27)		118.9(4)
C(23)-C(22)-C(3)		122.6(4)
C(27)-C(22)-C(3)		118.2(4)
C(22)-C(23)-O(26)	124.1(4)	
C(22)-C(23)-C(24)	119.5(4)	
O(26)-C(23)-C(24)	116.4(4)	
C(22)-C(23)-C(24)	119.5(4)	121.0(5)
C(25)-C(24)-C(23)	120.6(4)	119.0(5)
C(24)-C(25)-C(20)	121.2(4)	
C(26)-C(25)-C(24)		121.0(5)
C(25)-C(26)-C(27)		120.3(5)
C(26)-C(27)-C(22)		119.7(5)
C(29)-C(28)-C(7)	120.5(3)	120.9(4)
C(33)-C(28)-C(7)	120.4(3)	120.7(4)
C(29)-C(28)-C(33)	118.9(4)	118.4(4)
C(28)-C(29)-C(30)	120.4(4)	120.5(5)
C(31)-C(30)-C(29)	119.5(4)	120.6(5)
C(30)-C(31)-C(32)	120.1(4)	120.2(5)
C(31)-C(32)-C(33)	120.7(4)	119.7(5)
C(28)-C(33)-C(32)	120.3(4)	120.5(5)
Torsion Angles [°] (R- molecules)		
O(10)-C(10)-C(6)-C(5)	-32.7(6)	21.9(6)
Angles between planes [°]		
Center ring[a] – C5 phenyl ring[b]	89.49(12)	83.33(11)
Center ring[a] – C7 phenyl ring[c]	127.57(12)	74.09(14)
Center ring[a] – C3 phenyl ring[d]	55.68(12)	56.66(12)

a – S1,C2,C3,N4,C5,C6,C7,N8,C9
b – Phenyl ring bonded to C5
c – Phenyl ring bonded to C7
d – Phenyl ring bonded to C3
